# Outcomes of immunotherapy in medulloblastoma: a systematic review

**DOI:** 10.1093/oncolo/oyag109

**Published:** 2026-04-06

**Authors:** Patrícia Fontão, Marta Viana-Pereira, Filipa Pinto-Ribeiro, Thais Junqueira, Rui M Reis

**Affiliations:** Life and Health Sciences Research Institute (ICVS), School of Medicine, University of Minho, Braga 4710-057, Portugal; ICVS/3Bs-PT Government Associate Laboratory, Braga/Guimarães, Portugal; Life and Health Sciences Research Institute (ICVS), School of Medicine, University of Minho, Braga 4710-057, Portugal; ICVS/3Bs-PT Government Associate Laboratory, Braga/Guimarães, Portugal; Oncology Department, Hospital de Braga, Braga 4710-243, Portugal; Life and Health Sciences Research Institute (ICVS), School of Medicine, University of Minho, Braga 4710-057, Portugal; ICVS/3Bs-PT Government Associate Laboratory, Braga/Guimarães, Portugal; Barretos Children’s Cancer Hospital, Barretos 14784-390, São Paulo, Brazil; Life and Health Sciences Research Institute (ICVS), School of Medicine, University of Minho, Braga 4710-057, Portugal; ICVS/3Bs-PT Government Associate Laboratory, Braga/Guimarães, Portugal; Molecular Oncology Research Center, Barretos Cancer Hospital, Barretos 14784-400, São Paulo, Brazil

**Keywords:** medulloblastoma, pediatric brain tumor, immunotherapy, clinical trial outcomes

## Abstract

**Background:**

Medulloblastoma is the most common malignant brain tumor in children. Medulloblastoma has intrinsic characteristics that pose significant challenges to effective immunotherapy. Nevertheless, several clinical trials have explored immunotherapeutic strategies in patients diagnosed with medulloblastoma. This systematic review aimed to synthesize all immunotherapy modalities investigated in medulloblastoma patients and reported clinical outcomes.

**Methods:**

A systematic search was conducted in PubMed, Scopus, Web of Science, and ClinicalTrials.gov from inception to 30 June 2025 using the terms “immunotherapy” and (“brain tumor,” “pediatric brain tumor,” or “medulloblastoma”). Original articles, clinical trials, and conference abstracts evaluating any immunotherapeutic intervention in patients with medulloblastoma were included. Risk of bias was assessed using JBI critical appraisal tools.

**Results:**

Fifty-six studies met the inclusion criteria, encompassing at least 183 patients with medulloblastoma. Nearly half were Phase I trials (24/56, 43%), and 18% (10/56) were non-trial designs. Among the 29 studies reporting clinical outcomes, adoptive cellular therapies in combination regimens (7/29, 24%) and immune checkpoint inhibitors (6/29, 21%) were most frequently evaluated. Overall, clinical benefit was limited. Median overall survival ranged from 1.29 to 47 months, and median progression-free survival from 0.79 to 11 months. Progressive disease was reported in 40 patients, partial responses in 13, and complete responses in three patients.

**Conclusions:**

Despite increasing trial activity, immunotherapy has shown modest efficacy in patients with medulloblastoma. Interpretation is limited by small cohorts, heterogeneity, and inconsistent reporting of outcomes. Future studies should prioritize rational antigen selection, molecular subgroup stratification, and improved trial design.

Implications for Practice By systematically mapping published and ongoing clinical studies, this review clarifies the current clinical landscape of immunotherapy in medulloblastoma and indicates that, to date, clinical benefit remains limited. Clinicians should be aware that most available evidence is derived from small, early-phase, and highly heterogeneous studies, frequently enrolling heavily pretreated and relapsed patients, which restricts the generalizability of results. Future clinical practice should increasingly prioritize biomarker-driven patient selection and molecular subgroup stratification in order to improve the likelihood of therapeutic benefit, support realistic patient counseling, and better align immunotherapeutic strategies with the biological heterogeneity of medulloblastoma.

## Introduction

Medulloblastoma is the most common malignant brain tumor in children, accounting for approximately 20% of all pediatric central nervous system (CNS) tumors.^1^ The World Health Organization (WHO) classifies it as a grade IV malignancy, and its incidence is about 5 cases per 1 million individuals.^2^ Molecularly, these tumors are classified into four consensus molecular subgroups, which have been recognized by WHO since 2016.^3^^,^^4^ These four groups are Wingless (WNT), Sonic hedgehog (SHH), Group 3, and Group 4, which differ in demographics, histopathology, genetics, transcriptomics, epigenetics, proteomics, and clinical outcomes.^5^^,^^6^

Despite significant progress in understanding medulloblastoma biology, clinical management still relies on maximal safe surgical resection, craniospinal irradiation, and multi-agent chemotherapy, and the translation of molecular insights into targeted therapies has lagged behind.^5^^,^^6^ Although overall cure rates reach 70%-80%, conventional therapy is associated with significant long-term sequelae, including neurologic and cognitive impairments, endocrine dysfunction, infertility, neuropsychological deficits, and secondary malignancies.^5–7^ In addition to treatment-related morbidity, relapse and metastasis are major causes of mortality in medulloblastoma, with approximately 30% of patients failing conventional therapy or experiencing recurrence, often with metastatic dissemination.^5^^,^^6^ These patients typically respond poorly to salvage therapy, resulting in a median survival of less than 1 year.^2^^,^^7^^,^^8^ Indeed, recurrence accounts for 95% of all deaths in patients with medulloblastoma.^7^ Collectively, the limited therapeutic options for relapsed medulloblastoma and the adverse effects that compromise survivors’ quality of life highlight the urgent need for new therapies.

In this context, immunotherapy has emerged as a promising avenue, given its transformative impact on several adult solid tumors, particularly through immune checkpoint inhibitors.^9^^,^^10^ The most extensively studied targets are the cytotoxic T-lymphocyte-associated antigen 4 (CTLA-4) and programmed cell death protein 1 (PD-1).^11^ Monoclonal antibodies targeting these pathways are now routinely used as first-line therapies for multiple tumor types, following their initial approval by the U.S. Food and Drug Administration (FDA) in 2011 and 2014, respectively.^12^ In addition, multiple immunotherapeutic strategies have been explored in patients with medulloblastoma, including cancer vaccines, adoptive cell therapy, oncolytic viruses, and immunomodulatory agents.^13^^,^^14^ Medulloblastoma, however, presents unique challenges, such as low tumor mutational burden (TMB), limited immunogenicity, low microsatellite instability (MSI), and poor immune cell infiltration.^13–15^ Evidence addressing immunotherapy in medulloblastoma remains fragmented, often derived from early-phase trials with small patient numbers or mixed pediatric CNS tumor cohorts. To date, no comprehensive synthesis of clinical outcomes across the spectrum of immunotherapeutic modalities evaluated specifically in medulloblastoma has been conducted.

This systematic review aims to consolidate and critically evaluate the clinical evidence on immunotherapeutic interventions in medulloblastoma patients, summarizing treatment strategies, patient characteristics, clinical outcomes, and safety profiles. By mapping existing data and identifying knowledge gaps, this review seeks to inform future trial design and support the development of more effective and biologically rational immunotherapeutic approaches for patients with medulloblastoma.

## Materials and methods

This systematic review was conducted in accordance with PRISMA guidelines,^16^ and the review protocol was prospectively registered in PROSPERO (CRD420251048800; available at https://www.crd.york.ac.uk/PROSPERO/view/CRD420251048800).

### Search strategy

A comprehensive literature search was performed in four databases: ClinicalTrials.gov, PubMed, Scopus, and Web of Science. Search queries used the terms: “immunotherapy” AND (“brain tumor” OR “pediatric brain tumor” OR “medulloblastoma”).

Database queries were performed on the following dates: PubMed (May 21, 2025), Scopus (May 23, 2025), and ClinicalTrials.gov and Web of Science (June 30, 2025). Searches were restricted to publications in English. In addition to database searches, backward citation (reference list) screening of included articles was conducted to identify additional relevant studies and enhance the rigor of our systematic review.

### Eligibility criteria

Studies were selected based on the PICO framework. Population (P): patients diagnosed with medulloblastoma (any age). Intervention (I): Studies testing any immunotherapeutic intervention. Comparison (C): Not applicable; we included single-arm and non-comparative trials. Outcome (O): Key outcomes of interest included clinical response measures, including objective response rate (ORR), progression-free survival (PFS), overall survival (OS), adverse events, and tolerability.

Accordingly, we included interventional clinical trials regardless of phase. We also included single case reports, case series, and conference abstracts reporting clinical trial data, given the rarity of medulloblastoma and the limited availability of large studies. We excluded preclinical studies, reviews, and publications in languages other than English.

### Study selection and screening

All records retrieved were exported to Microsoft Excel and deduplicated. An initial manual screening of titles and abstracts was performed by one reviewer (P.F.) to assess relevance. Articles considered potentially eligible underwent full-text review. Final inclusion decisions were made by consensus between two reviewers (P.F. and M.V.P.); disagreements were resolved by discussion with the study team. No automation tools were used for screening.

### Data extraction and analysis

One researcher (P.F.) extracted the data into a standardized spreadsheet, and all items were independently verified by a second investigator (M.V.P.). No data collection automation tools were used. Where available, information on the study type, status, design, arms (if applicable), cohorts/groups, treatment plan, total number of included patients, total number of included medulloblastoma patients, clinical information for these patients, and outcomes (overall survival (OS), progression-free survival (PFS), time to progression, objective response rate (ORR), and adverse events) was extracted. No data was obtained or confirmed from the study authors, and only readily accessible data was extracted from publications and registry records.

### Quality assessment

Risk of bias and methodological quality were assessed using the Joanna Briggs Institute (JBI) critical appraisal tools appropriate to each design, including quasi-experimental studies (non-randomized clinical trials),^17^ case reports, and case series.^18^

### Data synthesis and statistical analysis

We summarized study characteristics and outcomes descriptively (pie charts, tables, and narrative synthesis). Given the heterogeneity of study designs, small sample sizes, varying outcome definitions and response criteria, and the predominance of non-randomized and single-arm studies, no formal meta-analysis was conducted.

## Results

### Study selection characteristics

**Figure 1.**
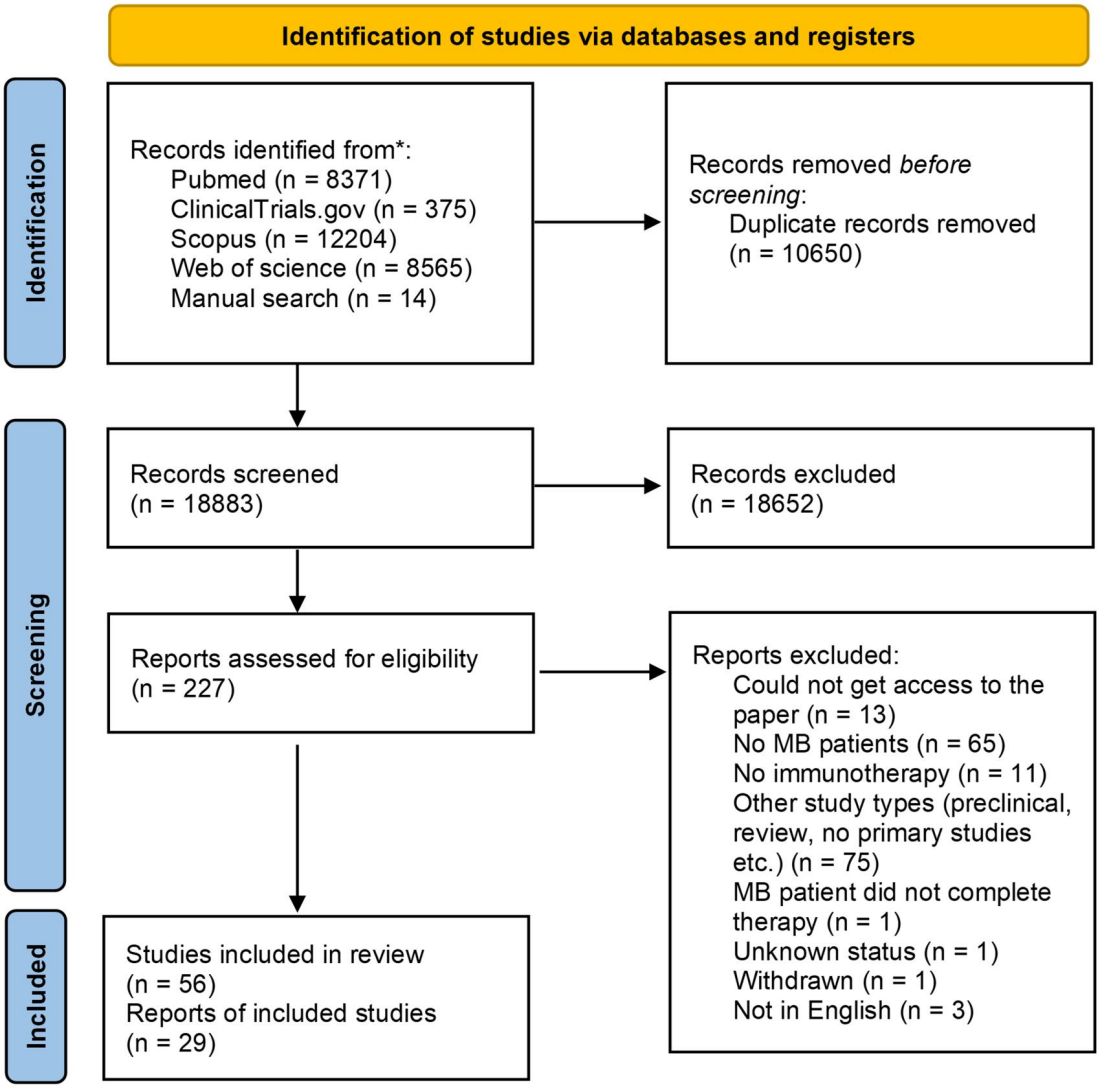
PRISMA flow diagram summarizing the study selection process. A total of 18883 records were identified, 227 full texts were screened for eligibility, 56 studies met the inclusion criteria, and 29 of these reported clinical outcomes.

Using the search strategy outlined above, we identified 227 potential studies across the four databases, including registered clinical trials from ClinicalTrials.gov. Following screening and application of eligibility criteria, 56 studies were included. However, only 29 had publicly available clinical results, reported in peer-reviewed publications or conference abstracts (Figure 1 and Tables 1; Tables S1-S4).

**Table 1.** Characteristics and reported outcomes of immunotherapy clinical studies involving MB patients.

Study	NCT	Type of study	Treatment	*n*	*n* MB	Age	Gender	Molecular subgroup	Dissemination	OS	PFS	ORR
**Adoptive cellular therapy and others**												
**Okamoto et al.^19^**		Case series	LAK+IL-2	6	6	6 (2-9)	Male, *n* = 4 Female, *n* = 2		Yes (CSF), *n* = 6		CR (20 months), *n* = 1 PD, *n* = 3	
**Ibayashi et al.^20^**		Case series	LAK+IL-2	9	1	56	Female, *n* = 1		Yes, *n* = 1			PD, *n* = 1
**Salmaggi et al.^21^**		Case series	α-IFN + r-IL-2	3	2	27 (20-34)	Male, *n* = 1 Female, *n* = 1		Yes, *n* = 2			PD, *n* = 2
**Silvani et al.^22^**		Case report	LAK+IL-2	1	1	>17	Female, *n* = 1		Yes (CSF), *n* = 1			CR/remission, *n* = 1
**Sankhla et al.^23^**		Case series	LAK+IL-2	10	R/R, *n* = 1	5	Male, *n* = 1		Yes (subarachnoid)	40 weeks (after immunotherapy)		PD, *n* = 1
**Thakar et al.^24^**	NCT02100891	Phase II trial. Conference abstract	Allogeneic HCT+ Donor NK Cell Infusion	15	1				All patients: 1-year OS: 64% 2-year OS: 40%	All patients: 1-year PFS: 29% 2-year PFS: 22%		
**Segal et al.^25^**	NCT01875601	Phase I trial. Conference abstract	NK cells + rhIL-15	16	R/R, n≥1	All patients: 16.1						PR, n≥1
**Immune checkpoint blockade**												
** Blumenthal et al.^26^**		Retrospective analysis	Pembrolizumab	22	R/R, *n* = 1	All patients: 5 (3-7)	Male, *n* = 1			All patients: 3.2 months (2.3-7.9)		PD, *n* = 1
**Gorsi et al.^27^**		Retrospective analysis	Nivolumab	10	R/R, *n* = 1	11	Female, *n* = 1		Yes (leptomeningeal) *n* = 2			PD, *n* = 1
** MarjaŃska et al.^28^**		Case series	Nivolumab	10	NIVO monotherapy (salvage therapy), *n* = 1	17.9	Male, *n* = 1					PD, *n* = 1
**Penas-Prado et al.^29^**	NCT03173950	Phase II trial. Conference abstract	Nivolumab	30	n≥ 1							SD (6 months)
**Geoerger et al.^30^**	NCT02332668	Phase I/II trial	Pembrolizumab	155	R/R (PD-L1+ tumors), *n* = 2	All patients: 13 years (IQR 8-15),				All patients: 9.0 months (95% CI 6.2-14.5)	All patients: 1.9 months (95% CI 1.8-1.9)	No effect
**Dunkel et al.^31^**	NCT03130959	Phase 1b/2 trial	Nivolumab + Ipilimumab	166	R/R, *n* = 30			Module A: SHH, *n* = 1, Group 3, *n* = 2, Group 4, *n* = 6, UN, *n* = 2, NE, *n* = 4 Module B: WNT, *n* = 1, SHH, *n* = 2, Group 3, *n* = 2, UN, *n* = 3, NE, *n* = 7	Module A: 7.4 (95% CI, 2.5-30.2) Module B: 22.2 (95% CI, 13.8-N.A.)	Module A: 1.4 (80% CI: 1.2-1.4) months Module B: 2.8 (80% CI: 1.5-4.5) months	Pseudoprogression, *n* = 1 (Module A)	
**Monoclonal antibodies and others**												
**Kramer et al. 2007^32^**		Phase I trial	^131^I-3F8	15	R/R, *n* = 4	9.5 (9-16)			Yes (leptomeningeal), *n* = 4 (w/ CSF *n* = 1)			No effect, *n* = 4
**Kramer et al. 2018^33^**	NCT00445965	Phase II trial	^124^I-3F8 or ^131^I-3F8	42	High-risk or R/R, *n* = 42	7 (2-43)				24.9 (16.3-55.8) months	11 (2.0-16.8) monthsSD, *n* = 9 Improvement/near resolution, *n* = 1 PD, *n* = 14 Free of disease, *n* = 15	
**Kramer et al. 2022^34^**	NCT00089245	Phase I trial	^131^I-Omburtamab	38	R/R, *n* = 6	All patients: 6.6 (1-2-53.5)					Non-NB: 0.2 years	Non-NB: PD, *n* = 6 SD, *n* = 5
**Small molecule inhibitors and others**												
**Johnson et al. 2024^35^**	NCT02502708	Phase I trial	Indoximod + chemotherapy +radiation	81	R/R, *n* = 13		SHH, *n* = 2 non-WNT/non-SHH, *n* = 5	Yes, *n* = 11 No, *n* = 2	All patients: 21.1 (0.4-61.9) months	CR, *n* = 1 PR, *n* = 2 LTS, *n* = 1		
**Johnson et al. 2023^36^**	NCT04049669	Conference abstract, Phase II trial	Indoximod + chemotherapy + radiation	53	R/R, *n* = 12					13.5 months		Patients with lesion response, *n* = 9/11
**Adoptive cellular therapy**												
**Khatua et al.^37^**	NCT02271711	Phase I trial	Autologous NK cells	9	R/R, *n* = 5	16 (8-18)	Male, *n* = 3 Female, *n* = 2		Yes (leptomeningeal), *n* = 4			PD, *n* = 5 (transient response, *n* = 1)
**Lin et al.^38^**	NCT04099797	Phase I trial	GD2-CAR-T cells	11	R/R GD2+, *n* = 2 DL1, *n* = 1 (2 infusions) DL2, *n* = 1 (3 infusions)	16 (14-18)		Non-WNT/non-SHH; MYC/MYCN not amp, *n* = 2				SD (at 6 weeks)
**Vaccines**												
**Olin et al.^39^**	NCT01171469	Phase I trial	DC vaccine	12	RR, *n* = 1 (dose level 3)	24	Female, *n* = 1					PD, *n* = 1
** Thompson et al.^40^**	NCT03299309	Phase I trial	PEP-CMV	42	R/R, *n* = 2			SHH, *n* = 2		1.29 (0.76-1.81) months	0.79 (0.76-0.82) months	
**Immunomodulatory**												
** Fangusaro et al. 2021a^41^**		Phase I trial	Pomalidomide	29	R/R, *n* = 2					All patients: 12 month OS: 28.1 +/- 8.4 %	All patients: 12 month PFS: 3.5 +/-2.4 %	PD, *n* = 2
** Fangusaro et al. 2021 b^42^**	NCT03257631	Phase II trial	Pomalidomide	53	R/R, *n* = 10	10 (4-17)	Male, *n* = 7 Female, *n* = 3			11.6 (95% CI: 1.74-NA) months	8.43 (95% CI: 7.29-18) weeks	Long-term SD: 0 % PD, *n* = 5 SD (3 cycles), *n* = 1 NE, *n* = 3
**Virus**												
**Yu et al.^43^**	NCT02962167	Phase I trial	Oncolytic measles virus (MV-NIS)	34	R/R, *n* = 29	9 (3-31)	Male, *n* = 18 Female, *n* = 5	WNT, *n* = 1 SHH, *n* = 2 Non-WNT/SHH, *n* = 11 NOS, *n* = 9			4 month PFS: 70% (47-100%, stratum C)	
**Virus and others**												
**Schuelke et al.^44^**	NCT02444546	Phase I trial	Wt Reovirus + Sargramostim (GM-CSF)	6	R/R, *n* = 1	17	Male			529 days		PD, *n* = 1
**Cytokines**												
** Hirakawa et al.^45^**		Case series	IFN-α	10	R/R, *n* = 2	10 (9-11)	Male, *n* = 1 Female, *n* = 1			47 (40-54) months	PR, *n* = 1 Unchanged, *n* = 2	
**Bacteria**												
**Selker et al.^46^**		Case series	Corynebacterium parvum administration	6	R/R (terminal), *n* = 1	17	Male, *n* = 1					
**Immune checkpoint blockade and others**												
**André et al.^47^**	NCT03585465	Phase I/II trial	Nivolumab + Chemotherapy	16	R/R, *n* = 2	13.5 (12-15)	Male, *n* = 1 Female, *n* = 1	WNT, *n* = 1 Group 4, *n* = 1	All patients: 6-month OS: 44% (95% CI: 20-66)	All patients: 3-month PFS: 37 (95% CI: 15-60) 6-month PFS: 12 (95%CI: 2-33)	PD, *n* = 2	

Abbreviations: CAR-T, chimeric antigen receptor T cell; CI, confidence interval; CR, complete response; CSF, cerebrospinal fluid; DC, dendritic cell; HCT, hematopoietic cell transplantation; IFN, interferon; IL, interleukin; IQR, interquartile range; LAK, lymphokine activated cells; LTS, long-term survivor; MB, medulloblastoma; MV-NIS, modified measles virus; NA, not applicable; NB, neuroblastoma; NE, not evaluable; NK, natural killer; PD, progressive disease; PEP-CMV, cytomegalovirus-specific peptide vaccine; PR, partial response; R/R, relapsed/refractory; SD, stable disease; Wt, wild type; UN, unknown.

**Figure 2.**
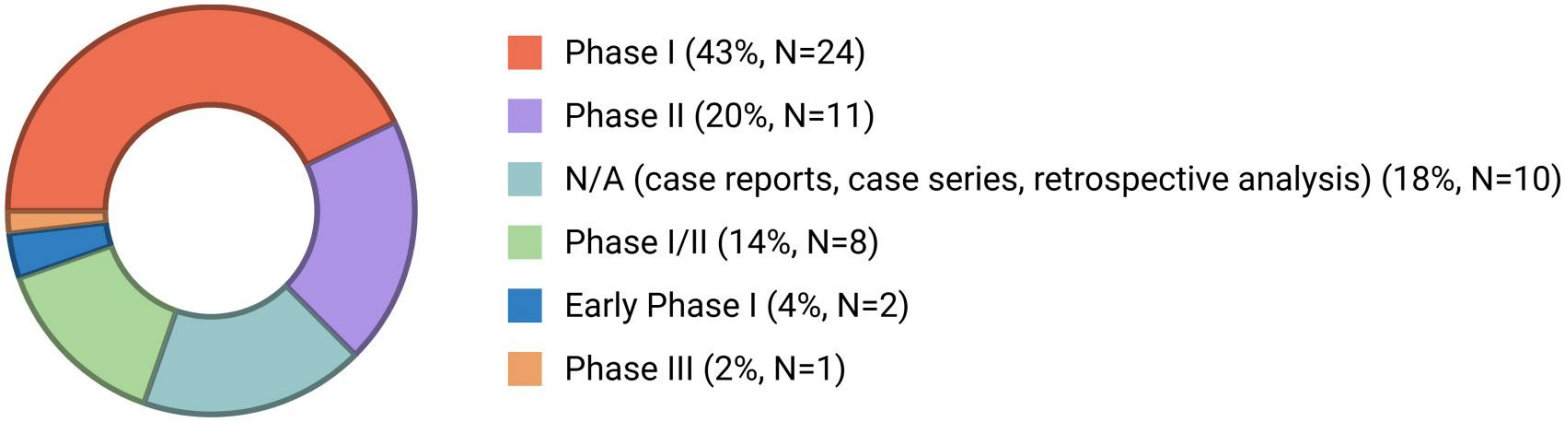
Distribution of included studies by study type and clinical trial phase. Created with BioRender.com.

The included studies comprised 46 clinical trials, 1 case report, 7 case series, and 2 retrospective analyses (case series). Overall, the evidence indicates that immunotherapy testing in medulloblastoma patients remains in its early stages, with approximately 50% of trials being Phase I studies, 4% early Phase I, and 18% not classified as clinical trials (Figure 2; Table S1 and S4).

### Immunotherapeutic modalities evaluated

Each study was categorized according to immunotherapy type, including immune checkpoint blockade, vaccines, adoptive cell therapy, small-molecule inhibitors, immunomodulatory agents, oncolytic viruses, cytokines, bacteria, monoclonal antibodies, and combination strategies. Although dendritic cell (DC) vaccines are technically a subtype of adoptive cell therapy, they were classified as vaccines to distinguish them from the infusion of activated lymphocytes.

**Figure 3.**
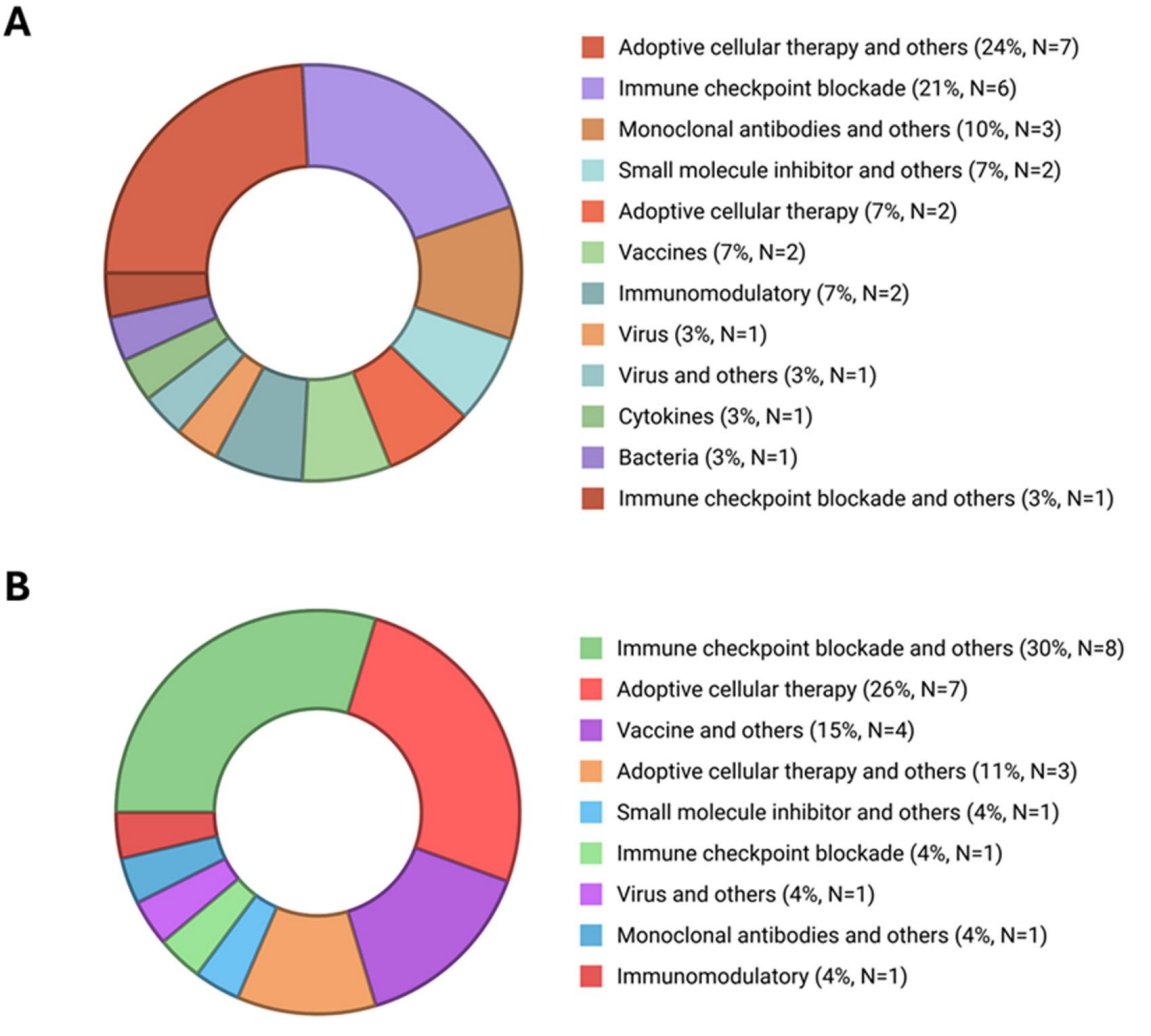
Distribution of immunotherapeutic modalities evaluated in medulloblastoma studies. (A) published studies with available results; and (B) ongoing, completed, or terminated studies without published outcomes. Created with BioRender.com.

To illustrate the immunotherapy landscape in the setting of medulloblastoma, studies with published results were analyzed separately from ongoing or completed trials without published outcomes (Figure 3). Among studies with available results, adoptive cellular therapies in combination with other strategies (7/29, 24%) and immune checkpoint blockade (anti-PD-1/PD-L1, 6/29, 21%) were the most represented (Figure 3A). In contrast, the current clinical trial landscape shows a shift toward immune checkpoint blockade combined with other strategies (8/26, 30%), followed by adoptive cellular therapies (7/26, 26%) (Figure 3B). Overall, these data highlight immune checkpoint blockade and adoptive cellular therapy as the most frequently explored immunotherapeutic interventions in patients with medulloblastoma.

### Patient demographics

The included studies were clinically and methodologically heterogeneous. Across all studies, a total of 183 patients diagnosed with medulloblastoma were reported (Tables 1; and Table S2). Several clinical characteristics were inconsistently reported, including age, gender, molecular subgroup, histologic classification, metastasis at study entry, prior therapies, follow-up time, and outcomes. All patients had recurrent, refractory, relapsed, high-risk, or disseminated disease (Table S2).

Age was reported for 114 patients, with a weighted median of 9 years (95% CI, 7–9). Gender was reported for 59 patients, of whom 42 were male and 17 were female. Molecular subgroup information was available for 57 patients, including three WNT, nine SHH, four Group 3, seven Group 4, 18 non-WNT/non-SHH, five unknown, and 11 non-evaluable. Histopathological classification was reported for 44 patients, including 29 classic, nine anaplastic/large cell, three primitive neuroectodermal tumors (PNET), two desmoplastic, and one medullomyoblastoma. Metastatic status was reported for 30 patients, of whom 25 had disseminated disease and 5 had no metastases.

### Quality assessment of the included studies

A formative quality assessment was performed using the Joanna Briggs Institute (JBI) critical appraisal tools appropriate for each study design (Table 2).

**Table 2.** Quality assessment of the selected studies based on JBI critical appraisal tools.

Study	JBI checklist	Items										Inclusion/exclusion
		1	2	3	4	5	6	7	8	9	10	
**Selker et al.^46^**	Case series	N	Y	Y	N	Y	Y	Y	Y	N	N/A	Inclusion
**Hirakawa et al.^45^**	Case series	Y	Y	Y	N	U	Y	Y	Y	U	N/A	Inclusion
**Okamoto et al.^19^**	Case series	U	Y	Y	Y	U	Y	Y	Y	Y	N/A	Inclusion
**Ibayashi et al.^20^**	Case series	U	Y	Y	U	U	Y	Y	Y	U	N/A	Inclusion
**Salmaggi et al.^21^**	Case series	U	Y	Y	U	U	Y	Y	Y	N	N/A	Inclusion
**Silvani et al.^22^**	Case report	Y	Y	Y	Y	Y	Y	N/A	Y	–	—	Inclusion
**Sankhla et al.^23^**	Case series	Y	Y	Y	Y	U	Y	Y	Y	Y	N/A	Inclusion
**Kramer et al. 2007^32^**	Quasi-experimental	Y	N	N/A	N/A	Y	N/A	U	U	N/A	—	Inclusion
**Olin et al.^39^**	Quasi-experimental	Y	N	N/A	N/A	Y	N/A	Y	Y	N/A	—	Inclusion
**Blumenthal et al.^26^**	Case series	Y	U	U	N	U	Y	Y	Y	N	N/A	Inclusion
**Kramer et al. 2018^33^**	Quasi-experimental	Y	N	N/A	N/A	Y	N/A	Y	Y	Y	—	Inclusion
**Gorsi et al.^27^**	Case series	Y	U	U	Y	Y	Y	Y	Y	Y	N/A	Inclusion
**Georger et al.^30^**	Quasi-experimental	Y	N	N/A	N/A	Y	N/A	Y	N	Y	—	Inclusion
**Kathua S et al.^37^**	Quasi-experimental	Y	N	N/A	N/A	Y	N/A	Y	Y	N/A	—	Inclusion
**MarjaŃska et al.^28^**	Case series	Y	U	U	Y	Y	Y	Y	Y	N	Y	Inclusion
**Thakar et al.^24^**	Quasi-experimental	Y	N	N/A	N/A	U	N/A	U	U	U	—	Inclusion
**Fangusaro et al., 2021a^41^**	Quasi-experimental	Y	N	N/A	N/A	Y	N/A	Y	Y	Y	—	Inclusion
**Fangusaro et al., 2021b^42^**	Quasi-experimental	Y	N	N/A	N/A	Y	N/A	Y	Y	Y	—	Inclusion
**Penas-Prado et al.^29^**	Quasi-experimental	Y	N	N/A	N/A	U	N/A	U	N/A	U	—	Inclusion
**Kramer et al. 2022^34^**	Quasi-experimental	Y	N	N/A	N/A	Y	N/A	Y	Y	Y	—	Inclusion
**Schuelke et al.^44^**	Quasi-experimental	Y	N	N/A	N/A	Y	N/A	Y	Y	N/A	—	Inclusion
**Dunkel et al.^31^**	Quasi-experimental	Y	N	U	U	Y	N/A	Y	Y	Y	—	Inclusion
**Johnson et al. 2023^36^**	Quasi-experimental	Y	N	N/A	N/A	U	N/A	Y	N/A	Y	—	Inclusion
**Segal et al.^25^**	Quasi-experimental	Y	N	N/A	N/A	Y	N/A	Y	N/A	U	—	Inclusion
**André et al.^47^**	Quasi-experimental	Y	N	N/A	N/A	Y	N/A	Y	Y	Y	—	Inclusion
**Johnson et al. 2024^35^**	Quasi-experimental	Y	N	N/A	N/A	Y	N/A	Y	U	Y	—	Inclusion
**Lin et al.^38^**	Quasi-experimental	Y	N	N	Y	Y	Y	Y	Y	Y	—	Inclusion
**Thompson et al.^40^**	Quasi-experimental	Y	N	N/A	N/A	N/A	N/A	Y	N/A	Y	—	Inclusion
**Yu et al.^43^**	Quasi-experimental	Y	N	N/A	N/A	Y	N/A	Y	Y	Y	—	Inclusion

Abbreviations: Y, yes; N, no; N/A, not applicable; U, unknown.

Several methodological limitations were identified across the included studies. For case reports and case series, the main concerns are unclear eligibility criteria and uncertainty about whether participants were consecutively and completely included. In most studies, these aspects were insufficiently described, limiting transparency and increasing the risk of selection bias. For non-randomized clinical trials (quasi-experimental studies), the major limitation was the absence of control groups, which precluded formal comparative analyses. Consequently, causal inferences regarding treatment efficacy remain limited. Additional concerns included small sample sizes, heterogeneous patient populations, and variable outcome reporting, further increasing the risk of bias and limiting generalizability.

Overall, the methodological quality of the evidence was moderate to low, which was expected and acceptable for case series/reports and early phase clinical trials.

### Treatment response and survival outcomes

Table 1 and Tables S1-S3 summarize the characteristics and reported outcomes of the included studies. Across the eligible trials, 20 immunotherapeutic approaches were evaluated in patients with medulloblastoma. These included indoximod (an IDO1 inhibitor) in combination with chemotherapy and/or radiotherapy in two studies; radiolabeled antibodies such as ^124^I-3F8 and ^131^I-3F8 in two studies and ^131^I-omburtamab in one study; adoptive cell therapies including natural killer (NK) cell infusion in three studies, with or without IL-15, and CAR-T cells in one study; lymphokine-activated killer (LAK) cells combined with IL-2 in four studies; immune checkpoint inhibitors (nivolumab, pembrolizumab, and ipilimumab) in six studies; immunomodulatory agents, particularly pomalidomide, in two studies; vaccination strategies using DCs or modified peptide vaccines (eg, PEP-CMV) in one study each; bacterial-based in one study and oncolytic viral therapies in two studies, including wild-type reovirus and modified measles virus; and cytokine IFN-α in one study.

As mentioned, a formal meta-analysis was not performed. Despite this, reported survival outcomes indicate median OS ranging from approximately 1.29 to 47 months, and median PFS ranging from 0.79 to 11 months across studies. Within the adoptive cellular therapy and others category, only one study reported survival data for a single patient diagnosed with medulloblastoma, with an OS of 40 weeks. No specific survival data of patients with medulloblastoma was available for immune checkpoint blockade. In the monoclonal antibodies and others category, a single study evaluating a radiolabeled anti-GD2 antibody reported a median OS of 24.9 (16.3-55.8) months and a median PFS of 11 (2.0-16.8) months.

For small molecule inhibitors and others categories, preliminary results from a Phase II trial testing indoximod in combination with chemotherapy and radiotherapy reported a median OS of 13.5 months. In the vaccine category, a Phase I trial evaluating a PEP-CMV vaccine reported a median OS of 1.29 (0.76-1.81) months and a median PFS of 0.79 (0.76-0.82) months. Regarding immunomodulatory agents, a Phase II trial of pomalidomide reported a median OS of 11.6 (95% CI: 1.74-NA) months and a median PFS of 8.43 (95% CI: 7.29-18) weeks. In the category of virus and others, a Phase I trial combining wild-type reovirus with sargramostim (GM-CSF) reported an OS of 529 days in one patient with medulloblastoma. Finally, in the cytokine category, a case series of two patients with medulloblastoma who were treated with IFN-α reported a median OS of 47 (40-54) months.

With respect to objective response, progressive disease was the most frequent outcome, documented in 40 patients (40/183, 21.9%) across 14 studies (14/29, 48.3%). Nevertheless, partial and complete responses were observed. Partial responses were reported in at least 13 patients, mostly among those receiving indoximod combined with chemotherapy and/or radiotherapy, but also in individuals treated with IFN-α (*n* = 1) or NK cell infusion with IL-15 (*n* ≥ 1). Complete responses were documented in three patients, two receiving LAK plus IL-2, and one treated with indoximod in combination with chemotherapy and/or radiotherapy.

Immune checkpoint inhibitors targeting PD-1, PD-L1, or CTLA-4 were evaluated in only seven studies (7/29, 24.1%), encompassing at least 38 patients, one of which combined immune checkpoint inhibitors with chemotherapy. These agents were primarily tested in patients with medulloblastoma that relapsed or were refractory and demonstrated minimal to no clinical activity. Median PFS and OS could not be determined across these trials due to the lack of individual patient survival data. Objective responses were not reported, with most patients (*n* = 5) experiencing progressive disease, and only a single case of stable disease was reported. In the study by Dunkel et al.^31^ which evaluated nivolumab as monotherapy and in combination with ipilimumab, objective responses for patients with medulloblastoma were not clearly reported. However, disease progression was observed in 88.6% of the overall cohort, strongly suggesting that patients with medulloblastoma most likely experienced progressive disease. No complete or partial responses were observed, highlighting the challenges of checkpoint blockade in patients diagnosed with medulloblastoma (Table 1 and Table S3).

Taken together, while isolated cases of tumor response were evident, the predominance of disease progression and the variability in study design emphasize the need for more robust, controlled clinical trials to clarify the therapeutic potential of immunotherapies in patients diagnosed with medulloblastoma.

### Safety and adverse events

Analysis of adverse events observed in patients with medulloblastoma was substantially limited by the lack of specific reporting across the included studies (Table S3). In most trials, adverse event data were presented only for the overall study cohort rather than stratified by tumor type, preventing the extraction of adverse event frequency or severity for patients with medulloblastoma. Only 11 studies, encompassing 72 patients diagnosed with medulloblastoma, reported specific adverse events. Adverse events in clinical trials were generally graded using the National Cancer Institute Common Terminology Criteria for Adverse Events (CTCAE) version 4 or 5.

Among studies reporting adverse events, the most commonly described events of any grade included headache (at least 6/72), nausea (at least 3/72), vomiting (at least 6/72), fever (5/72), fatigue (at least 3/72), neutropenia (4/72), thrombocytopenia (3/72), leukopenia (3/72), and cytokine release syndrome (CRS) (2/72). Neurological toxicities were also noted, including CSF pleocytosis/chemical meningitis (1/72), bilateral Lasègue sign (1/72), stupor (1/72), transient hallucinations (1/72), tremor (1/72), insomnia (1/72), hydrocephalus (1/72), semicomatose state (1/72), and increased intracranial pressure (1/72).

Overall, the adverse events reported were broadly consistent with known toxicities of immunotherapies,^48^ including hematologic abnormalities, flu-like symptoms, immune-related dermatologic or gastrointestinal events, and occasional neurologic complications. However, due to heterogeneous and non-granular reporting, it was not possible to estimate adverse event incidence or severity among patients with medulloblastoma nor to compare safety across treatment modalities. Consequently, the available evidence does not allow for a reliable safety profile for immunotherapy in patients with medulloblastoma, underscoring the need for standardized, tumor-specific adverse events reporting in future trials.

Overall, across diverse immunotherapeutic strategies, clinical benefit in patients diagnosed with medulloblastoma was limited, with disease progression representing the predominant outcome and only sporadic partial or complete responses observed.

## Discussion

Over recent decades, immunotherapy and particularly immune checkpoint blockade have transformed the treatment of several cancer types.^9^^,^^10^ More recently, adoptive cellular therapies, such as CAR-T cells, have generated significant interest due to their success in hematologic malignancies.^49–51^ In contrast, evidence supporting immunotherapy in patients diagnosed with medulloblastoma remains limited and fragmented. We therefore conducted this systematic review to consolidate and critically evaluate all published and ongoing studies assessing any form of immunotherapy in medulloblastoma patients. By synthesizing evidence spanning several decades of clinical development, we provided a comprehensive overview of the immunotherapeutic strategies explored in this disease and identified common challenges and future directions. Overall, the available evidence does not support a consistent or clinically meaningful benefit of immunotherapy in patients with medulloblastoma to date.

Across the 56 included studies, comprising clinical trials, case series, case reports, and retrospective analyses, our findings indicate that immunotherapy for patients with medulloblastoma remains in its infancy. Most clinical trials were early-phase or Phase I studies. Evaluation of treatment modalities revealed an evolution over time. Older studies most frequently explored combinations of adoptive cellular therapy with IL-2,^19^^,^^20^^,^^22^^,^^23^ followed by immune checkpoint blockade targeting PD-1/PD-L1.^26–31^^,^^47^ In contrast, current and ongoing trials increasingly focus on combining immune checkpoint inhibitors with radiation or other agents and on next-generation cellular therapies, including CAR-T cells targeting GD2, HER2, and B7-H3 (Table S4). This shift likely reflects both advances in immunotherapy technology and the limited clinical benefit observed in earlier trials, prompting the exploration of more potent or rational combination strategies. Indeed, progressive disease was the predominant response reported (40 patients across 14 studies), with only isolated cases of partial or complete responses. Among the more encouraging signals were studies combining indoximod with chemotherapy and/or radiation. In a Phase I trial (NCT02502708), one patient experienced a complete response, two experienced partial responses, and one patient remained a long-term survivor, among the 13 participants.^35^ These results led to an ongoing Phase II trial (NCT04049669), where preliminary data show lesion responses in 9 of 11 participants.

The limited clinical benefit of immunotherapy in patients with medulloblastoma is thought to be associated with several intrinsic features of this tumor type. In contrast to most solid tumors in adults, pediatric brain tumors are characterized by low TMB and, consequently, a reduced neoantigen repertoire, as well as low MSI.^13–15^^,^^52^ Regarding PD-1/PD-L1 expression in medulloblastoma, several studies reported that expression levels are typically low, with occasional tumors exhibiting higher expression.^53–58^ Consistent with these observations, our group reported similar findings and demonstrated a distinct immune checkpoint profile of tumors from patients diagnosed with medulloblastoma.^59^ Moreover, medulloblastoma is associated with a relatively immune-cold tumor microenvironment (TME), characterized by limited infiltration of cytotoxic T cells and enrichment of myeloid-derived immune populations,^55^^,^^60–62^ which may further restrict effective antitumor immunity. Emerging evidence also suggests that immune landscape features vary across molecular subgroups, highlighting the complexity of the TME in this disease.^54^^,^^55^^,^^60^^,^^63^^,^^64^

Nevertheless, there is a subset of patients with medulloblastoma associated with constitutional mismatch repair deficiency (cMMRD), characterized by an ultrahypermutated genotype driven by defects in DNA mismatch repair pathways, resulting in a very high neoantigen burden, that may benefit from immune checkpoint blockade.^65–67^ To the best of our knowledge, only one study has described a patient with cMMRD-associated medulloblastoma treated with a combination of nivolumab and chemotherapy, which was initiated following the detection of colonic polyps.^67^ Therefore, current evidence remains insufficient to establish a clear association between cMMRD status and sensitivity of medulloblastoma to immune checkpoint blockade. Nonetheless, such findings illustrate that immunotherapy may be highly effective in selected molecular contexts of pediatric brain tumors,^65^^,^^67–69^ and systematic molecular characterization and biomarker-driven patient selection will likely be critical for identifying those patients who may derive the greatest benefit from immunotherapy.

Importantly several limitations must be acknowledged. First, substantial heterogeneity existed across study designs, as we included early-phase clinical trials alongside case series and case reports. Moreover, all clinical trials were non-randomized, non-blinded, and lacked control groups. While acceptable in exploratory studies, these methodological features introduce a high risk of bias. Second, most trials enrolled mixed CNS malignancy cohorts rather than only patients diagnosed with medulloblastoma, reflecting the disease rarity (incidence ∼5 per million^2^), and inclusion/exclusion criteria differed regarding prior therapies, surgical resection, molecular characteristics, and other factors, which pose challenges for recruitment and feasibility. Third, several studies had an extremely small number of patients, with 12 out of 29 studies reporting data from only a single patient with medulloblastoma. Altogether, this heterogeneity limits direct comparison between studies. Fourth, the majority of patients had recurrent, relapsed, refractory, or high-risk disease, often heavily pretreated, making clinical outcomes difficult to interpret and not readily generalizable. Off-label or salvage treatment used in patients with advanced disease further contributed to heterogeneity. Importantly, recurrent medulloblastoma tumors are genetically and biologically distinct from the primary disease,^6^^,^^70–73^ and most of the current knowledge regarding TME is derived from diagnostic specimens.^55^^,^^60^^,^^63^^,^^74^ A recent study demonstrated that the immune landscape of relapsed tumors differs from that observed at diagnosis, highlighting important temporal changes in the TME during disease progression.^75^ As prior therapies and tumor recurrence profoundly reshape the immune microenvironment,^76–78^ this represents another critical knowledge gap and a limitation of the current immunotherapy clinical trial landscape. Nevertheless, synthesizing this heterogeneous body of evidence remains valuable, as it highlights recurring biological and methodological challenges in immunotherapy for patients with medulloblastoma and helps identify priorities for the design of future biomarker-driven clinical trials.

Another important limitation was the inadequate use of molecular subgrouping, which was expected in earlier studies in the field. Only six studies reported subgroup classification, despite established differences in prognosis and tumor biology across WNT, SHH, Group 3, and Group 4 medulloblastoma.^5^^,^^6^ Emerging evidence also indicates subgroup-specific differences in the tumor immune microenvironment.^55^^,^^60^^,^^63^^,^^74^ Furthermore, although several trials tested targeted agents, including PD-1/PD-L1 inhibitors, anti-GD2, and anti-B7-H3, only two studies confirmed target expression prior to therapy. Given that medulloblastoma tumors typically exhibit low PD-L1 expression and modest T-cell infiltration,^53^^,^^54^^,^^56^^,^^59^ such biomarker-agnostic inclusion limits interpretability. Similar concerns apply to B7-H3. Although we have recently demonstrated elevated B7-H3 expression in tumors from patients with medulloblastoma compared with normal cerebellum,^79^ treatment with a radiolabeled anti-B7-H3 monoclonal antibody (^131^I-omburtamab) was associated with only modest clinical benefit. Among patients without the diagnosis of neuroblastoma, the median PFS was 0.2 years, and most individuals experienced stable or progressive disease.^34^ Nonetheless, despite the modest clinical benefit, a follow-up study (NCT04167618) with ^177^Lu-DTPA-omburtamab was initiated but terminated due to business priorities. Moreover, a Phase I trial (NCT04185038) testing B7-H3.CAR-T cells is currently enrolling patients.

Another major limitation relates to safety reporting. Adverse events were inconsistently described across studies, and very few trials stratified adverse events specifically for patients with medulloblastoma. As a result, we were unable to determine the frequency, severity, or spectrum of toxicities attributable to immunotherapies in this population.^5–7^ Moreover, as previously mentioned, most included patients had relapsed or refractory disease who had received multiple prior therapies, which complicates attribution of adverse events and limits the ability to distinguish treatment-related effects from cumulative toxicity of prior therapies.

Lastly, due to the limited clinical benefit, follow-up time, and adverse events, it is not possible to draw reliable conclusions regarding long-term sequelae, which is a critical consideration for patients with medulloblastoma, given the known neurocognitive and endocrine vulnerabilities associated with CNS-directed treatments in the pediatric population. This underscores the need for standardized adverse event reporting and long-term follow-up measures tailored to patients diagnosed with medulloblastoma in future immunotherapy trials.

Looking ahead, several priorities emerge for advancing immunotherapy for patients diagnosed with medulloblastoma. Future studies should incorporate molecular subgroup stratification and standardized eligibility criteria, as biological heterogeneity likely contributes to variable therapeutic responses. Improved reporting standards, particularly for adverse events, prior treatments, and immunologic biomarkers, are needed to enable cross-trial comparisons and strengthen evidence synthesis. There is also a clear need for early-phase trials to integrate correlative immune profiling, including assessment of TME, immune infiltrates, and target antigen expression to rationalize patient selection. Combination strategies that address the unique challenges of medulloblastoma, including its immune-cold microenvironment, low neoantigen burden, and the anatomic constraints of the CNS, together with improved selection of biomarkers for each patient profile, may hold the greatest promise. Finally, collaborative multicenter designs will be essential to overcome limited patient numbers and accelerate progress in this rare pediatric malignancy.

## Conclusions

Current clinical evidence indicates that immunotherapy has demonstrated limited and inconsistent clinical benefit in patients diagnosed with medulloblastoma. Interpretation is constrained by marked heterogeneity in study design, small cohort of patients, nonrandomized approaches, and frequent inclusion of mixed CNS tumor populations.

These findings highlight the urgent need for more standardized and biologically rational clinical trials for patients diagnosed with medulloblastoma, incorporating molecular subgroup stratification, biomarker-driven, harmonized outcome measures, and tumor-specific safety reporting. Despite these limitations, this systematic review provides the most comprehensive and up-to-date synthesis of immunotherapy studies in patients with medulloblastoma and offers a framework to guide future translational and clinical research efforts.

## Supplementary Material

oyag109_Supplementary_Data

## Data Availability

Data supporting the findings of this study are available within the article and its supplementary materials.
