# Supplementary material for: Outcomes of immunotherapy in medulloblastoma: a systematic review
Source: Oncologist. 2026 Apr 6;31(6):oyag109. doi: 10.1093/oncolo/oyag109 (PMC13195807; doi:10.1093/oncolo/oyag109)
Supplement: oyag109_Supplementary_Data [file oyag109_supplementary_data.docx]

**Supplementary table 1.** Detailed study characteristics of the included studies (with results).

| **Study** | **NCT** | **Type of study** | **Status** | **Study design** | **Treatment** | **Cohorts/groups** | **Treatment plan** |
| --- | --- | --- | --- | --- | --- | --- | --- |
| Johnson et al., 2024 | NCT02502708 | Phase I trial | Completed | Non-randomized, single-arm, parallel assignment, open-label, two-institution | Indoximod +Chemotherapy (temozolomide, metronomic cyclophosphamide, etoposide) + Radiation | Group 1: Dose finding Indoximod + temozolomide Group 2: Recurrent expansion cohort: Indoximod (RP2D) + temozolomide; ± indoximod RP2D) + radiation Group 3a: Dose-finding Indoximod + radiation Group 3b: newly diagnosed DIPG expansion cohort: Indoximod (RP2D) + radiation followed by Indoximod (RP2D) + temozolomide Group 4: Indoximod + metronomic cyclophosphamide + etoposide | Indoximod: 19.2 mg/kg/dose twice daily, 28 days Temozolomide: 200 mg/m^2^/day, 5 days Metronomic cyclophosphamide: 2.5 mg/kg orally once daily Etoposide: 50 mg/m^2^ orally once daily for 21 days |
| Kramer et al., 2018 | NCT00445965 | Phase II trial | Completed | Single-center, single-group assignment, open-label | ^124^I-3F8 or ^131^I-3F8 |  | Initial dose: 2 mCi + 2 mg of ^124^I-3F8 or ^131^I-3F8 (week 1) Follow-up dosing: Weekly 10 mCi + 2 mg ^131^I-3F8 for up to 4 injections |
| Khatua S et al 2020 | NCT02271711 | Phase I trial | Completed | Dose-escalation, 3+3 design, single-group assignment | Autologous NK cells | Dose level 1 Dose level 2 Dose level 3 | Dose level 1: 1×10⁶/m² per infusion, 3 infusions/week (cumulative 9×10⁶/m²/cycle) Dose level 2: 1×10⁷/m² per infusion, 3 infusions/week or 3×10⁷/m² once weekly (cumulative 9×10⁷/m²/cycle) Dose level 3: 3×10³/m² per infusion, 1 infusion/week (cumulative 9×10⁹/m²/cycle) |
| Salmaggi A, et al., 1994 |  | Case series |  |  | α-IFN + r-IL-2 |  | Recombinant α-IFN: 1.2 million IU, twice daily, 5 days per cycleRecombinant IL-2: 0.9 million IU, twice daily, 5 days per cycleIndomethacin: 50 mg i.m., twice daily during treatmentCycle schedule: Each treatment cycle followed by a 3-week washout period |
| Okamoto Y, et al., 1988 |  | Case series |  |  | One-haplotype identical LAK + IL-2 |  | LAK cells: 2–10 × 10⁸ cells, 2–3x/week Recombinant IL-2: 25–50U, 2–3x/week Route: Intrathecal via Ommaya reservoir or ventriculo-peritoneal shunt |
| Ibayashi Y, et al., 1993 |  | Case series |  |  | LAK + IL-2 |  | LAK cells: 5.9 × 10⁷ 2-3 times Recombinant IL-2: 200U 2-3 times Route: Intrathecal |
| Kramer K, et al., 2007 |  | Phase I trial | Completed |  | ^131^I-3F8 | Dose level 1: 10mCi Dose level 2: 20mCi | Initial tracer: 1–2 mCi for dosimetry Therapy injection 4–7 days later Premedication: acetaminophen, morphine, lorazepam, diphenhydramine |
| Sankhla SK, et al., 1996 |  | Case series |  |  | LAK + IL-2 |  | LAK cells 2.5×10⁶ per instillation × 4–5 instillations (total 10×10⁶) |
| MarjaŃska A, et al., 2020 |  | Case series |  | Off-label, salvage therapy | Nivolumab + Ipilimumab |  | Monotherapy: Nivolumab 3 mg/kg every 2 weeks Combination therapy: Nivolumab 3 mg/kg + Ipilimumab 1 mg/kg every 3 weeks for 4 doses, followed by Nivolumab 3 mg/kg every 2 weeks (adjuvant phase) |
| Blumenthal DT, et al., 2016 |  | Retrospective analysis |  | Off-label, salvage therapy | Pembrolizumab |  | 50 mg every 3 weeks (in all children median of 4 infusions (2-10)) |
| Fangusaro J, et al., 2021 a |  | Phase I trial |  | Multicenter, dose escalation | Pomalidomide | Dose finding groups: -Dose level 1 (1.9mg/m2), n=6-Dose level 2, (2.6mg/m2), n=18-Dose level 3, (3.4mg/m2), n=5 | Pomalidomide: 0.5–4 mg, daily ×21 days, 28-day cycle  Route: Oral |
| Kramer K, et al., 2022 | NCT00089245 | Phase I trial | Terminated (Corporate business decision. No safety or efficacy concerns.) | Single-center, 3+3 dose escalation and expansion design, Single Group Assignment, open-label | ^131^I-omburtamab |  | Dose escalation: all patients received one dose of ^131^I-omburtamab Therapeutic: 370 MBq (10 mCi) 36 patients received therapeutic dose (n=19 received 1 dose, n=17 received 2 doses) Route: intraventricular |
| Gorsi HS, et al., 2019 |  | Retrospective analysis |  | Off-label, compassionate use | Nivolumab |  | Nivolumab: 3 mg/kg, IV, every 2 wk, 3 doses |
| Olin MR, et al., 2014 | NCT01171469 | Phase I trial | Completed | Single institution, dose escalation | DC vaccine | Dose Level 1 (DL1): 5 × 10⁶ DCs + Imiquimod  Dose Level 2 (DL2): 10 × 10⁶ DCs + Imiquimod  Dose Level 3 (DL3): 15 × 10⁶ DCs + Imiquimod | GBM6-AD/DC vaccine: 5–15 ×10⁶ cells, every 2 wk, post-debulking surgery |
| Hirakawa K, et al., 1983 |  | Case series |  |  | IFN-α |  | IFN therapy: 5 ×10⁴–3 ×10⁶ IU, IM, 1–3x/week, 4–17 mo |
| Selker RG, et al., 1978 |  | Case series |  |  | *Corynebacterium parvum* administration |  | *C. parvum*: 5 mg/m² + Lasix: 40 mg, IV |
| Johnson TS, et al., 2023 | NCT04049669 | Conference abstract, interim results of a Phase II trial | Recruiting | Non-randomized, open-label, crossover assignment | Indoximod + chemotherapy (temozolomide, metronomic cyclophosphamide, etoposide) + radiation | Experimental Core Regimen: Sub-cohort A: Indoximod PO BID + Temozolomide PO Days 1–5 per cycle Sub-cohort B: Up-front Indoximod + partial radiation → Core Regimen chemo-immunotherapy Sub-cohort C: Up-front Indoximod + full-dose radiation (>50 Gy brain, >45 Gy spine) → Core Regimen chemo-immunotherapy  Experimental Salvage Regimens: Salvage 1: Indoximod + oral metronomic cyclophosphamide + etoposide Days 1–21 per cycle Salvage 2: Indoximod + oral lomustine Day 1 + Temozolomide per cycle | Indoximod: 8.4 mg/kg/day PO + Temozolomide: 200 mg/m²/day ×5 days, 28-day cycle; given during radiation if planned |
| Silvani A, et al., 1994 |  | Case report |  |  | LAK + IL-2 |  | LAK cells: 700 ×10⁶ + rIL-2: 3.2 ×10⁶ IU, IT via lumbar puncture, 3x/week, 2 cycles ×3 wk, 7-mo interval |
| Yu B, et al., 2025 | NCT02962167 | Phase I trial | Completed | Non-randomized, multicenter, open-label, parallel assignment, dose escalation 3+3 | MV-NIS oncolytic virus | Stratum A: Local recurrence, contrast-positive, CSF cytology negative  Stratum B: Disseminated recurrence  Stratum C: Disseminated MB recurrence | MV-NIS: 1×10⁷–1×10⁹ TCID₅₀, intracavity (Stratum A) or IT via LP (Stratum B), repeat dosing days 0 & 7 (Stratum C) |
| Lin FY, et al., 2024 | NCT04099797 | Phase I trial | Recruiting | Non-randomized, single-institution, open-label, parallel assignment, 3+3 design, | GD2-CAR-T cells | Dose level 0: 1 x 10^7^ cells/m2 of GD2.CARTs Dose level 1: 1 x 10^7^ cells/m2 C7R-GD2.CARTs Dose level 2: 3 x 10^7^ cells/m2 C7R-GD2.CARTs | GD2.CARTs ± C7R: 1–3 ×10⁷ cells/m² IV, 1–4 cycles, ≥6-wk intervals; lymphodepletion: fludarabine 30 mg/m² ×3d + cyclophosphamide 500 mg/m² ×2d |
| Segal J, et al., 2023 | NCT01875601 | Phase I trial. Conference abstract | Completed | Non-randomized, single-institution, open-label, parallel assignment, 3+3 design | NK cells + rhIL-15 | Cohort A. NK cell infusion (dose escalation) Cohort B. NK cell infusion + escalating doses of rhIL15 | Cohort A: NK cells 1×10⁶–1×10⁸ cells/kg IV, Day 0 post 2d cyclophosphamide Cohort B: NK cells 1×10⁷ cells/kg + rhIL-15 0.25–2 µg/kg/day IV ×10 |
| Penas-Prado M, et al., 2021 | NCT03173950 | Phase II trial. Conference abstract | Completed | Non-randomized, multi-institution, open-label, parallel assignment | Nivolumab |  | Nivolumab: 240 mg IV q2wk ×2 cycles, then 480 mg IV q4wk (up to 14 doses) |
| Thompson EM, et al., 2025 | NCT03299309 | Phase I trial | Active, not recruiting | single-arm, open-label, single-institution | PEP-CMV (peptide vaccine directed at pp65) |  | Temozolomide: 150–200 mg/m², Days 1–5; PEP-CMV vaccine: 250 µg/m² ID, admixed with Montanide, Day 21, repeat q2wk ×2, then monthly, every other dose for 2 yrs; Td preconditioning Day 20 MB patients: only 1 vaccine |
| Dunkel IJ, et al., 2023 | NCT03130959 | Phase 1b/2 trial | Completed | Multi-institutional, Non-randomized, parallel assignment, open-label, sequential-arm | Nivolumab ± Ipilimumab | Cohort 1: Newly diagnosed DIPG/H3K27M within 4 wks post-RT  Cohort 2: Recurrent/progressive non-brain stem HGG (including GBM) post-surgery + RT ± chemo  Cohort 3: Relapsed/resistant MB after ≥1 therapy line  Cohort 4: Relapsed/resistant ependymoma after ≥1 therapy line  Cohort 5: Other rare/complex high-grade CNS malignancies, recurrent/progressive post ≥1 therapy line | Module A: Nivolumab 3 mg/kg IV Q2W  Module B: Nivolumab 3 mg/kg + Ipilimumab 1 mg/kg IV q3wk ×4, then Nivolumab 3 mg/kg IV Q2W |
| Geoerger B, et al., 2020 | NCT02332668 | Phase I/II trial | Recruiting | Multi-institutional, Single-arm, Non-randomized, single group assignment, open-label, modified 3+3 design, | Pembrolizumab |  | Pembrolizumab: 2 mg/kg IV q3wk (established at Phase I) |
| Fangusaro J, et al., 2021 b | NCT03257631 | Phase II trial | Completed | Open-label, single-group assignment, multicenter | Pomalidomide |  | POM 2.6 mg/m^2^/day on days 1-21 of a 28-day cycle (up to 24)  Median of cycles for MB patients: 2 (range 1-4)  Median of treatment days for MB patients: 57.0 (range, 28-118) |
| André N, et al., 2024 | NCT03585465 | Phase I/II trial | Active, not recruiting | Multicenter, open-label, non-randomized, parallel assignment | Nivolumab + chemotherapy (vinblastine, cyclophosphamide, capecitabine) | Arm A: Nivolumab+ vinblastine+ cycophosphamide Arm B: Nivolumab+capecitabine Arm C: Nivolumab+vinblastine+cyclophosphamide+capecitabine | Nivolumab: 3 mg/kg IV Days 1 & 15 q28d (Arms A–C) Arm A: Vinblastine 2 mg/m² IV weekly + Cyclophosphamide 30 mg/m²/day PO (Days 1–4, 8–11, 15–18, 22–25) Arm B: Capecitabine 400–600 mg/m²/day PO Arm C: Vinblastine 2 mg/m² IV weekly + Cyclophosphamide 30 mg/m²/day (Days 1–4 & 15–18) alternating with Capecitabine 400–600 mg/m²/day (Days 8–11 & 22–25) Duration: Up to 2 years |
| Schuelke MR, et al., 2022 | NCT02444546 | Phase I trial | Completed | Single-institution, single group assignment, open-label, 3+3 design | Wild-type Reovirus + Sargramostim (GM-CSF) |  | GM-CSF: 250 µg/m² SC Days 1–2; Pelareorep: 3×10⁸ TCID₅₀ IV ×3 days; q28d cycles × up to 12 |
| S. Thakar M, et al., 2020 | NCT02100891 | Phase II. Conference abstract | Terminated (15 patients enrolled out of planned 20, terminated due to slow enrollment at end of study.) | Single-institution, open-label, single-group assignment | Allogeneic HCT + Donor NK Cell Infusion |  | Haplo-BMT + NK cells: RIC (fludarabine + cyclophosphamide + 3 Gy TBI), NK cells on Day +7 post-transplant |

**Supplementary table 2**. Patient characteristics of the included studies (with results).

| **Study** | ***n*** | ***n* MB** | **Age** | **Gender** | **Molecular subgroup** | **Histology** | **Metastatic dissemination** | **Previous therapy** |
| --- | --- | --- | --- | --- | --- | --- | --- | --- |
| Johnson et al., 2024 | 81  Group 1, n=21 Group 2, n=33 Group 3a, n=14 Group 3b, n=13 | R/R, n=13  Group 1, n=3 Group 2, n=7 Group 3a, n=3 |  |  | SHH, n=2 non-WNT/non-SHH, n=5 |  | Yes, n=11 No, n=2 | Surgical resection or debulking Radiation or proton therapy Systemic therapy Temozolomide Immunotherapy Autologous stem cell infusion |
| Kramer et al., 2018 | 42  4 full-dose, n=18 3 full-dose, n=13 2 full-dose, n=6 1 full-dose, n=6 | High-risk or R/R, n=42 | At diagnosis: 5 (1-33)  At beginning: 7 (2-43) |  |  | Classic, n=29 Anaplastic, n=8 PNET, n=3 Desmoplastic, n=1 Medullomyoblastoma, n=1 |  | Craniospinal radiation Myeloablative chemotherapy |
| Khatua S et al 2020 | 9 Dose level 1, n=3 Dose level 2, n=3 Dose level 3, n=3 | R/R, n=5 Dose level 1, n=2 Dose level 2, n=2 Dose level 3, n=1 | At beginning:16 (8-18) | Male, n=3 Female, n=2 |  |  | Leptomeningeal dissemination, n=4 |  |
| Salmaggi A, et al., 1994 | 3 | 2 | At diagnosis: 20.5 (12-29)  At beginning: 27 (20-34) | Male, n=1 Female, n=1 |  |  | Yes, n=2 | Chemotherapy (CDDP, BCNU, Ara-C+MTX) radiotherapy immunotherapy |
| Okamoto Y, et al., 1988 | 6 | 6 | At beginning: 6 (2-9) | Male, n=4 Female, n=2 |  |  | Yes, CSF dissemination, n=6 | Surgery, whole-neuraxis irradiation and chemotherapy (VCR, MTX, ACNV, ACNU) |
| Ibayashi Y, et al., 1993 | 9 | 1 | At beginning: 56 | Female, n=1 |  |  | Yes, n=1 |  |
| Kramer K, et al., 2007 | 15 Dose level 1, n=9 Dose level 2, n=6 | R/R, n=4 Dose level 1, n=3 Dose level 2, n=1 | At beginning: 9.5 (9-16) |  |  |  | Leptomeningeal disease, n=4 CSF cytology Yes, n=1 No, n=3 | Surgery, Chemotherapy, Radiothearpy, Myeloablative chemotherapy with stem-cell rescue |
| Sankhla SK, et al., 1996 | 10 | R/R, n=1 | At beginning: 5 | Male, n=1 |  |  | Yes, subarachnoid metastasis | Surgery and radiation |
| MarjaŃska A, et al., 2020 | 10 | NIVO monotherapy, n=1 | At beginning: 17.9 | Male, n=1 |  |  |  | CBDCA + VP-16 + VCR + CTX RTH, VCR+CPPD+CCNU |
| Blumenthal DT, et al., 2016 | 22 | Recurrent, n=1 | At beginning  All patients: 5 (3-7) | Male |  |  |  |  |
| Fangusaro J, et al., 2021 a | 29 | R/R, n=2 |  |  |  |  |  |  |
| Kramer K, et al., 2022 | 38 | R/R, n=6 | At beginning  All patients: 6.6 (1-2-53.5) |  |  |  |  | Yes (at least one prior regimen of chemotherapy and radiotherapy) |
| Gorsi HS, et al., 2019 | 10 | R/R, n=1 | At beginning: 11 | Female, n=1 |  |  | Yes, leptomeningeal dissemination | XRT, vincristine, cyclophosphamide, cisplatin, avastin, irinotecan, temozolomide, everolimus, vorinostat |
| Olin MR, et al., 2014 | 12 | RR, n=1 (dose level 3) | At beginning: 24 | Female, n=1 |  |  |  | Yes |
| Hirakawa K, et al., 1983 | 10 | R/R, n=2 Low-dose group, n=1 High-dose group, n=1 | At beginning :10 (9-11) | Male, n=1 Female, n=1 |  |  |  | Craniotomy and radiation |
| Selker RG, et al., 1978 | 6 | R/R (terminal), n=1 | At beginning: 17 | Male, n=1 |  |  |  | CCNU-VCR Procarbozine Medrola |
| Johnson TS, et al., 2023 | 53 | R/R, n=12 |  |  |  |  |  |  |
| Silvani A, et al., 1994 | 1 | 1 | At diagnosis: 12  At beginning: > 17 | Female, n=1 |  |  | Yes (CSF) | Surgical removal, radiotherapy, intrathecal chemotherapy (cis-diammino-dichloro-platinum, 45 mg/m^2^; etoposide, 120 mg/m^2^ for 2 days every month over a 4-month period) |
| Yu B, et al., 2025 | 34 | R/R, n=29 Stratum A, n=44 Stratum B, n=10 Stratum C, n=15 | At beginning: 9 ( 3-31) | Male, n=18 Female, n=5 | WNT, n=1 SHH, n=2 Non-WNT/SHH, n=11 NOS, n=9 | Desmoplastic, n=1 Large cell anaplasia, n=1 |  |  |
| Lin FY, et al., 2024 | 11 | R/R GD2+, n=2 DL1, n=1 (2 infusion cycles) DL2, n=1 (3 infusion cycles) | At beginning: 16 (14-18) |  | Non-WNT/non-SHH; *MYC*/*MYCN* not amp |  |  | Yes, chemotherapy (multiple regimens) and radiation |
| Segal J, et al., 2023 | 16 | R/R, n>1 | At beginning  All patients: 16.1 |  |  |  |  |  |
| Penas-Prado M, et al., 2021 | 30 | n> 1 |  |  |  |  |  |  |
| Thompson EM, et al., 2025 | 42 | R/R, n=2 |  |  | SHH (*TP53* wt), n=2 |  |  |  |
| Dunkel IJ, et al., 2023 | 166 Module A, n=85 Module B, n=81 Cohort 1, n=45 Cohort 2, n=31 Cohort 3, n=30 Cohort 4, n=22 Cohort 5, n=38 | R/R, n=30  Module A, n=15 Module B, n=15 |  |  | Module A:  SHH, n=1; Group 3, n=2: Group 4, n=6 UN, n=2; NE, n=4 Module B:  WNT, n=1; SHH, n=2; Group 3, n=2; UN, n=3; NE, n=7 |  |  | Yes, standard therapy |
| Geoerger B, et al., 2020 | 155 | R/R (PD-L1+ tumors), n=2 | At beginning  All patients: 13 years (IQR 8–15) |  |  |  |  |  |
| Fangusaro J, et al., 2021 b | 53 | R/R, n=10 | At beginning: 10 (4-17) | Male, n=7 Female, n=3 |  |  |  | Surgery (n=9), Radiation (n=9), Systemic therapy (n=9) |
| André N, et al., 2024 | 16 Arm A, n=3 Arm B, n=6 Arm C, n=7 | R/R, n=2 Arm A, n=1 Arm B, n=1 | At beginning: 13.5 (12-15) | Male, n=1 Female, n=1 | WNT (*APC* mutation), n=1 Group 4, n=1 |  |  | Yes |
| Schuelke MR, et al., 2022 | 6 | R/R, n=1 | At beginning: 17 | Male, n=1 |  |  |  | Yes, surgery, radiotherapy and chemotherapy |
| S. Thakar M, et al., 2020 | 15 | 1 |  |  |  |  |  |  |

**Supplementary table 3**. Detailed outcomes of the included studies (with results).

| **Study** | **Follow-up period** | **AEs** | **OS** | **PFS** | **ORR** |
| --- | --- | --- | --- | --- | --- |
| Johnson et al., 2024 | All patients: 52 (39-77) months | Extensive list reported only at the global study population level. | All patients: 21.1 (0.4-61.9) months |  | CR, n=1 PR, n=2 LTS, n=1 |
| Kramer et al., 2018 |  | Acute: Grade 2 or 3 fever, Headache, Nausea, Vomiting, Transient acute bradycardia with solomnence (2/42), Fatigue, CSF pleocytosis/chemical meningitis (1/42) Long-term: Moyamoya syndrome (1/42), Symptomatic small-vessel angiopathy (1/42), Meningioma, Ovarian fibrothecoma and basal cell carcinoma (1/42) (not directly attributed to ^131^I-3F8) | 24.9 (16.3-55.8) months **Estimates:** Six-month OS 88.1% (73.7-94.9%) 1-year OS 78.6% (62.9-88.2%) 5-year OS 44.9% (29.0-59.5%) | 11 (2.0-16.8) months **Estimates:** Six-month PFS 57.1% (40.9-70.4%) 1-year PFS 47.6% (32.1-61.6%) 5-year PFS 23.7% (11.7-38.0%) | W/radiographically evaluable disease: -SD, n=9 -Improvement/near resolution, n=1 -PD, n=9  Without radiographically evaluable disease: - Free of disease, n=15 -PD, n=5 |
| Khatua S et al 2020 | 30 days after last infusion | All patients: Grade 1: Dysmetria, dysarthria, Headache, Facial droop, Thrombocytopenia Grade 2: Sinus tachycardia, Headache, Dysphagia, Slurred speech, Fatigue, Anorexia, Thrombocytopenia Grade 3: Headache, Seizure, Fatigue |  |  | PD, n=5 (transient radiographic response, n=1) |
| Salmaggi A, et al., 1994 |  | Fever (2/2), bilateral Lasègue (1/2), headache (1/2), moderate tachycardia (1/2), stupor (1/2), transient hallucinations (1/2), tremors (1/2), insomnia (1/2) |  |  | PD, n=2 |
| Okamoto Y, et al., 1988 |  | Hydrocephalus (1/6) |  |  | CR (20 months), n=1 PD, n=3 |
| Ibayashi Y, et al., 1993 |  | None |  |  | PD, n=1 |
| Kramer K, et al., 2007 |  | Headache (4/4), nausea (2/4), vomiting (3/4), fever (2/4), leg pain (1/4), bone pain (1/4) (grade 1 or 2) |  |  | No effect |
| Sankhla SK, et al., 1996 |  | All patients: Hemiparesisi, focal seizures, headache, vomiting, local sepsis, fever (not graded) | 40 weeks (after immunotherapy) |  | PD, n=1 |
| MarjaŃska A, et al., 2020 |  | None |  |  | PD, n=1 |
| Blumenthal DT, et al., 2016 |  | All patients: Mild rash, increased liver enzymes, protracted diarrhea (only in 2 patients) | All patients: 3.2 months (2.3-7.9) |  | PD |
| Fangusaro J, et al., 2021 a |  | All patients: Grade 1: Lymphopenia, leucopenia, neutropenia, thrombocytopenia, anemia, fatigue, headache, hypocalcemia, alanine aminotransferase increased, skin and subcutaneous disorders, hypokalemia, constipation, diarrhea, vomiting, hyperkalemia, hypophosphatemia, metabolism and nutrition disorders Grade 2: lymphopenia, leucopenia, neutropenia, thrombocytopenia, anemia, fatigue, headache, hypocalcemia, alanine aminotransferase increased, hypoalbuminemia, skin and subcutaneous tissue disorders, constipation, vomiting, hyperkalemia, hypophosphatemia Grade 3: lymphopenia, leucopenia, neutropenia, thrombocytopenia, anemia, fatigue, headache, diarrhea Grade 4: lymphopenia, neutropenia, thrombocytopenia | All patients: 12 month OS: 28.1 +/- 8.4 % | All patients: 12 month PFS: 3.5 +/-2.4 % | PD, n=2 |
| Kramer K, et al., 2022 |  | All patients: Grade 1: fever, vomiting, anxiety, pruritus, ALT, bilirubin, AST, platelets, ANC Grade 2: hypertension, vomiting, hypothermia, ALT, AST, platelets, ANC, fever, headache Grade 3: AST/ALT elevation, chemical meningitis, nausea, dehydration, platelets, ANC, lymphopenia Grade 4: thrombocytopenia, neutropenia, platelets, ANC |  | Neuroblastoma: 11.1 years Non-neuroblastoma: 0.2 years | Non-NB: PD, n=6 SD, n=5 |
| Gorsi HS, et al., 2019 |  | All patients: Grade 2: leukopenia (3), transaiminitis (1), hyperglycemia (1), hypoalbuminemia (1), pancreatitis (1), anemia (1), nausea/vomiting (1), and thrombocytopenia (1). |  |  | PD, n=1 |
| Olin MR, et al., 2014 |  | None |  |  | PD, n=1 |
| Hirakawa K, et al., 1983 |  | All patients: Transient fever (in 2 patients in the high-dose group) | 47 (40-54) months |  | PR, n=1 Unchanged, n=1 |
| Selker RG, et al., 1978 |  | Fever, chills, ataxia, dysarthria, respiratory embarassment, semicomatose, increase in intracranial pressure |  |  |  |
| Johnson TS, et al., 2023 | Median 23 months (range 0.2-35.5) | ‒ | 13.5 months |  | Patients with lesion response, n=9/11 |
| Silvani A, et al., 1994 | 30 months | ‒ |  |  | CR/remission, n=1 |
| Yu B, et al., 2025 |  | All patients: Stratum B: ALT increased, lymphocite count decreased Stratum C: Upper respiratory infection, neutrophil count decreased  No higher than grade 3. |  | 4 month PFS: 70% (47-100%, stratum C) |  |
| Lin FY, et al., 2024 |  | Cytokine release syndrome, n=2 (grade 4, n=1) Tummor inflammation-associated neurotoxicity, n=2 |  |  | SD (at 6 weeks) |
| Segal J, et al., 2023 |  | All patients: Hematologic toxicity, CRS, pericardial tamponade and capillary leak syndrome |  |  | PR, n>1 |
| Penas-Prado M, et al., 2021 |  | All patients: Grade 3: Anemia, hydrocephalus, lymphopenia Grade 2: cerebral edema, headache |  |  | SD (6 moths) |
| Thompson EM, et al., 2025 |  | Grade 1: anemia (1/2) Grade 2: hypothermia (1/2) Grade 3: encephalopathy (1/2) | 1.29 (0.76-1.81) months | 0.79 (0.76-0.82) months |  |
| Dunkel IJ, et al., 2023 | Module A: 8.1 (range, 0.2–41.7) Module B: 10.8 (range, 0.7–34.7) | All patients: Fatigue, Decreased appetite, ALT increased, Abdominal pain, AST increased, Neutrophil count decreased, Headache, Diarrhea, WBC count decreased, Vomiting, Nausea, Rash, Weight decreased, Neurological disorders | Module A: 7.4 (95% CI, 2.5–30.2) Module B: 22.2 (95% CI, 13.8-not available [N.A.])  **12-month OS** Module A: 38.9% (95% CI, 14.3%–63.2%) Module B: 86.7% (95% CI, 56.4%– 96.5%) | Module A: 1.4 (80% CI: 1.2-1.4) months Module B: 2.8 (80% CI: 1.5-4.5) months  **6-month PFS** Module A: 0% Module B: 20.0% (95% CI, 4.9%–42.4%) | Pseudoprogression, n=1 (Module A) |
| Geoerger B, et al., 2020 | All patients: 8.6 months (IQR 2.5–16.4) | All patients: Grade 1-2: fatigue, anemia, pyrexia, decreased lymphocyte count, increased aspartate aminotransferase, hypothyroidism, nausea, rash, diarrhoea, abdominal pain, increased ALT, asthenia, hyperthyroidism, decreased white blood cell count, decreased appetite, pruritus, rash, decreased platelet count, arthralgia, erythema, colitis, decreased neutrophil count, pleural effusion, pneumonitis, pulmonary oedema, hypertension, dyspnoea Grade 3: anaemia, decreased lymphocyte count, increased aspartate aminotransferase, pruritus, colitis, gastric ulcer, decreased neutrophil count, hypertension, photosensitivity reaction, dyspnoea Grade 5: pleural effusion, pneumonitis, pulmonary oedema | All patients: 9.0 months (95% CI 6.2–14.5) | All patients: 1.9 months (95% CI 1.8–1.9) | No effect |
| Fangusaro J, et al., 2021 b |  | Any grade: Neutropenia (4/10), Leukopenia (3/10), Thrombocytopenia (3/10), Anemia (2/10), Alanine aminotransferase level increased (2/10), Constipation (2/10), maculopapular rash (2/10), pruritus (1/10), fatigue (2/10), decreased appetite (2/10), vomiting (2/10) Grade 3/4: neutropenia (3/10), hypokalemia (1/10) | 11.6 (95% CI: 1.74-NA) months | 8.43 (95% CI: 7.29-18) weeks | Long-term SD: 0 % PD, n=5 SD (3 cycles), n=1 NE, n=3 |
| André N, et al., 2024 |  | All patients: Arm A: lymphopenia Arm B: anaemia, lymphopenia, asthenia, venous access device related infection, hypokalaemia Arm C: anaemia, lymphopenia, thrombocytopenia, presyncope, asthenia, decreased appetite | All patients: 6-month OS: 44% (95% CI: 20-66) | All patients: 3-month PFS: 37 (95% CI: 15-60) 6-month PFS: 12 (95%CI: 2-33) | PD, n=2 |
| Schuelke MR, et al., 2022 |  | All patients Grade 1: anemia, fatigue, fever, flu-like symptoms, ALT increased, AST increased, lymphocyte count decreased, plaetelet count decreased, hypoalbuminemia, hypocalcemia, hypoglycemia, hypokalemia, hypomagnesemia, bone pain, headache Grade 2: dysphagia, nausea, vomiting, fatigue, flu-like symptoms, ALT increased, neutrophil count decreased, white blood cell count decreased, hyperglycemia, seizure Grade 3:lymphocyte count decreased, neutrophil count decreased, white blood cell count decreased, hyponatremia, hypophosphatemia, depressed level of consciousness Grade 4: confusion | 529 days |  | PD, n=1 |
| S. Thakar M, et al., 2020 | 1.3 years (range, 70 days – 5 years) | All patients: Acute graft-versus-host disease (GVHD), grade 2, n=2 Chronic GVGD, n=2 | All patients: 1-year OS: 64% 2-year OS: 40% | All patients: 1-year OS: 29% 2-year OS: 22% |  |

**Supplementary table 4.** Detailed information of the ongoing clinical trials or without reported results.

| **NCT identifier** | **Study title** | **Phase** | **Status** | **Conditions** | **Sponsor** | **Allocation** | **Interventional model** | **Masking** | **Participant group/arm** | **Intervention/treatment** | **Study start date** | **Study completion date** |
| --- | --- | --- | --- | --- | --- | --- | --- | --- | --- | --- | --- | --- |
| NCT05106296 | Repurposing Ibrutinib for Chemo-Immunotherapy in a Phase 1b Study of Ibrutinib With Indoximod Plus Metronomic Cyclophosphamide and Etoposide for Pediatric Patients With Brain Cancer | Phase I | Recruiting | Progressive or refractory: Ependymoma MB GBM Primary Brain Tumor | Theodore S. Johnson | N/A | Single group assignment | None (Open Label) | Experimental: Treatment Regimen (4-drug chemo-immunotherapy regimen, cycles are a minimum of 28 days). | Ibrutinib: oral, once daily (days 1–21)  Indoximod: oral, twice daily  Cyclophosphamide: oral, once daily (days 1–21)  Etoposide: oral, once daily (days 1–21) | 08/02/2022 | 2026-09 |
| NCT06193759 | Immunotherapy for Malignant Pediatric Brain Tumors Employing Adoptive Cellular Therapy (IMPACT) | Phase I | Recruiting | MB, Childhood ATRT of CNS Embryonal Tumor With Multilayered Rosettes Pineoblastoma Embryonal Tumor of CNS | Children's National Research Institute | N/A | Single group assignment | None (Open Label) | Experimental: Embryonal brain tumors, young children (<5 years of age) with newly diagnosed high-risk embryonal CNS malignancies. | Multi-TSA-T directed against proteogenomically determined personalized TSA | 20/09/2024 | 29/12/2032 |
| NCT05298995 | Phase I Study of Anti-GD2 Chimeric Antigen Receptor-Expressing T Cells in Pediatric and Young Adult Patients Affected by Relapsed/Refractory Central Nervous System Tumors | Phase I | Recruiting | Brain Tumor, Pediatric MB, Childhood Embryonal Tumor High Grade Glioma Diffuse Midline Glioma DIPG Brain Tumor Adult | Bambino Gesù Hospital and Research Institute | Non-Randomized | Single Group Assignment | None (Open Label) | Arm A: MB/other embryonal tumor Arm B: Hemispheric HGG Arm C: Thalamic HGG, DMG, DIPG and other rare CNS tumors not included in Arms A and B | GD2-CART01 (iC9-GD2-CAR T-cells): single IV infusion at a dose of 1.0–6.0 × 10⁶ cells/kg, following lymphodepleting chemotherapy | 09/11/2023 | 2038-11 |
| NCT03911388 | Phase 1 Trial of Engineered HSV G207 in Children With Recurrent or Refractory Cerebellar Brain Tumors | Phase I | Recruiting | Progressive or refractory: MB, GBM multiforme, giant cell GBM, anaplastic astrocytoma, PNET, ependymoma, ATRT, germ cell tumor, other high-grade malignant tumor | M.D. Anderson Cancer Center | N/A | Single group assignment (A traditional 3 + 3 design will be used with four patient cohorts) | None (Open Label) | Experimental: HSV G207 | G207: intratumoral infusion via stereotactic catheters; if safe, followed by 5 Gy focal radiotherapy within 24 h. | 12/09/2019 | 1/09/2027 |
| NCT02359565 | A Safety and Preliminary Efficacy Trial of Pembrolizumab (MK-3475) in Children With Recurrent, Progressive or Refractory Diffuse Intrinsic Pontine Glioma (DIPG), Non-Brainstem High-Grade Gliomas (NB-HGG), Ependymoma, Medulloblastoma or Hypermutated Brain Tumors | Phase I | Active, not recruiting | CMMRD, Lynch Syndrome, Malignant Glioma  Recurrent or refractory: Brain Neoplasm, Childhood Ependymoma,DIPG, MB | National Cancer Institute (NCI) | N/A | Single group assignment | None (Open Label) | Experimental: Pembrolizumab | Pembrolizumab: IV, every 21 days (up to 34 cycles) | 03/06/2015 | 21/12/2025 |
| NCT01326104 | Recurrent Medulloblastoma and Primitive Neuroectodermal Tumor Adoptive T Cell Therapy During Recover From Myeloablative Chemotherapy and Hematopoietic Stem Cell Transplantation | Phase II | Completed | MB Neuroectodermal Tumor | University of Florida | Non-Randomized | Parallel assignment | None (Open Label) | Group A: High dose chemotherapy plus peripheral blood stem cell transplant followed by TTRNA-xALT and TTRNA-DCs. Group B: NMA Salvage chemotherapy plus peripheral blood stem cell transplant followed by TTRNA-xALT and TTRNA-DCs. | TTRNA-xALT: 3 × 10⁷/kg administered once IV.  TTRNA-DCs: 1 × 10⁷ cells administered ID every 2 weeks, for a total of 3 doses. | 07/09/2010 | 28/03/2025 |
| NCT03500991 | Phase 1 Study of HER2-Specific CAR T Cell Locoregional Immunotherapy for HER2 Positive Recurrent/Refractory Pediatric Central Nervous System Tumors | Phase I | Active, not recruiting | HER2-positive: Central Nervous System Tumor, Pediatric; Glioma; Ependymoma; MB; Germ Cell Tumor; ATRT; PNET; Choroid Plexus Carcinoma; Pineoblastoma | Seattle Children's Hospital | Non-Randomized | Parallel assignment | None (Open Label) | Arm A (tumor cavity infusion): CAR T cells administered in patients with supratentorial tumors.  Arm B (ventricular system infusion): CAR T cells administered in the fourth or lateral ventricle in patients with infratentorial or leptomeningeal tumors. | HER2-CAR T cells: autologous CD4/CD8 T cells, lentivirally transduced, administered via indwelling CNS catheter. | 26/07/2018 | 26/07/2039 |
| NCT07017816 | JACOB: Joint Administration of cDNA for TP53, checkpOint Inhibition and Boost/Hypofractionated Radiation. A Phase 0/1 Study in Children With Recurrent, Progressive or Refractory CNS Malignancies. | Early phase I | Not yet recruiting | CNS Malignancies MB Recurrent ATRT Recurrent Pineoblastoma Embryonal Neoplasm Ependymoma Recurrent High Grade Gliomas | Children's National Research Institute | N/A | Single group assignment | None (Open Label) | Experimental: Treatment Arm Patients with recurrent, progressive, or refractory CNS malignancies | Drug: SGT-53  Radiation: hypofractionated radiotherapy with immunotherapy  Drug: Nivolumab | 2025-07 | 01/05/2028 |
| NCT04185038 | Phase 1 Study of B7-H3-Specific CAR T Cell Locoregional Immunotherapy for Diffuse Intrinsic Pontine Glioma/Diffuse Midline Glioma and Recurrent or Refractory Pediatric Central Nervous System Tumors | Phase I | Recruiting | CNS Tumor; DIPG; Diffuse Midline Glioma; Ependymoma; MB, Childhood; Germ Cell Tumor; ATRT; PNET; Choroid Plexus Carcinoma; Pineoblastoma, Childhood; Glioma | Seattle Children's Hospital | Non-Randomized | Parallel Assignment | None (Open Label) | Arm A: Local infusion into resection cavity; non-DIPG supratentorial tumors.  Arm B: Intraventricular infusion; non-DIPG infratentorial or leptomeningeal tumors.  Arm C: Intraventricular infusion; DIPG tumors. | SCRI-CARB7H3(s) / B7H3-CAR T cells: autologous CD4⁺/CD8⁺ T cells expressing B7H3-CAR and EGFRt, administered via indwelling CNS catheter. | 11/12/2019 | 2041-05 |
| NCT05768880 | Phase 1 Study of B7-H3, EGFR806, HER2, And IL13-Zetakine (Quad) CAR T Cell Locoregional Immunotherapy For Pediatric Diffuse Intrinsic Pontine Glioma, Diffuse Midline Glioma, And Recurrent Or Refractory Central Nervous System Tumors | Phase I | Recruiting | DIPG; Diffuse Midline Glioma; Recurrent CNS Tumor, Adult; Recurrent, CNS Tumor, Childhood; Refractory Primary Malignant Central Nervous System Neoplasm | Seattle Children's Hospital | Non-Randomized | Parallel assignment | None (Open Label) | Arm A: DIPG Arm B: DMG & recurrent/refractory tumors | SC-CAR4BRAIN: autologous CAR T cells administered intraventricularly once weekly for 3 consecutive weeks, followed by 1 week off. | 05/05/2023 | 31/12/2043 |
| NCT04730349 | Phase 1/2 Study of Bempegaldesleukin in Combination With Nivolumab in Children, Adolescents, and Young Adults With Recurrent or Refractory Malignancies (PIVOT IO 020) | Phase I/II | Terminated (Business objectives have changed) | Ependymoma Ewing Sarcoma High-grade Glioma Leukemia and Lymphoma MB Miscellaneous Brain Tumors Miscellaneous Solid Tumors NB Relapsed, Refractory Malignant Neoplasms Rhabdomyosarcoma | Bristol-Myers Squibb | Non-Randomized | Sequential assignment | None (Open Label) | Part A (dose-escalation): Four dosing schedules (A1W, A1F, A2W, A2F).  Part B (disease-specific expansion cohorts):  B1 neuroblastoma; B2 Ewing sarcoma; B3 rhabdomyosarcoma; B4 miscellaneous solid tumors; B5 NHL/leukemia; B6 high-grade glioma; B7 MB and other embryonal tumors; B8 ependymoma; B9 miscellaneous brain tumors. | Nivolumab: specified dose on specified days.  NKTR-214: specified dose on specified days. | 03/06/2021 | 22/06/2022 |
| NCT05096481 | Phase 2 Trial of a Novel Peptide Vaccine (PEP-CMV) Targeting CMV Antigen for Newly Diagnosed Pediatric High-grade Glioma and Diffuse Intrinsic Pontine Glioma and Recurrent Medulloblastoma | Phase II | Recruiting | High Grade Glioma DIPG Recurrent MB | Nationwide Children's Hospital | N/A | Single group assignment | None (Open Label) | Experimental: PEP-CMV | PEP-CMV vaccine: 250 µg/m² (max 500 µg) + Montanide ISA-51 1:1, ID on day 21; first 3 doses every 2 weeks, then monthly, up to 24 cycles.  Temozolomide: 200 mg/m²/day orally, Days 1–5 of Cycle 1.  Td vaccine: Booster at enrollment; preconditioning 6–24 h before first PEP-CMV dose. | 18/07/2024 | 15/06/2030 |
| NCT06396481 | Clinical Study of Allogeneic Vγ9Vδ2 T Cells in the Treatment of Brain Malignant Glioma | Early phase I | Not yet recruiting | GBM DIPG Brain Tumor MB | Beijing Tiantan Hospital | Non-Randomized | Parallel Assignment | Quadruple (Participant, Care Provider, Investigator, Outcomes Assessor) | Experimental: Vγ9Vδ2 T cells | Vγ9Vδ2 T cells: intraventricular administration via Ommaya reservoir, dose-escalation (1×10⁷, 3×10⁷, 1×10⁸, 3×10⁸ cells), followed by an expansion phase at the selected dose; one cycle every 4 weeks for 3 cycles, with optional radiotherapy or chemotherapy during week 4. | 30/04/2024 | 30/12/2027 |
| NCT03690869 | A Safety and Pharmacokinetic Study of Single Agent REGN2810 in Pediatric Patients With Relapsed or Refractory Solid or Central Nervous System (CNS) Tumors and a Safety and Efficacy Trial of REGN2810 in Combination With Radiotherapy in Pediatric Patients With Newly Diagnosed Diffuse Intrinsic Pontine Glioma, Newly Diagnosed High-Grade Glioma, or Recurrent High-Grade Glioma | Phase I/II | Terminated (Sponsor Decision) | Relapsed Solid Tumor Refractory Solid Tumor Relapsed CNS Tumor Refractory CNS Tumor DIPG High Grade Glioma | Regeneron Pharmaceuticals | Randomized | Parallel assignment | None (Open Label) | Experimental: Phase 1 Patients in both the Solid Tumor Cohort and the CNS Cohort will receive cemiplimab monotherapy. Each Cohort will have 2 subgroups by age (0 to <12 years, 12 to <18 years). Experimental: Efficacy with Newly Diagnosed DIPG Experimental: Efficacy with Newly Diagnosed HGG Experimental: Efficacy with Recurrent HGG ≥ 3 to < 12 years cohort and 12 to ≤ 25 years cohort with combination of cemiplimab and radiation therapy | Cemiplimab: IV, as monotherapy (Phase 1) or combined with radiation, continued as maintenance.  Radiation: Conventional or hypofractionated, combined with IV cemiplimab. | 24/09/2018 | 10/05/2023 |
| NCT03696030 | A Phase 1 Cellular Immunotherapy Study of Intraventricularly Administered Autologous HER2-Targeted Chimeric Antigen Receptor (HER2-CAR) T Cells in Patients With Brain and/or Leptomeningeal Metastases From HER2 Positive Cancers | Phase I | Active, not recruiting | Malignant Neoplasm Metastatic Malignant Neoplasm in the Brain Metastatic Malignant Neoplasm in the Leptomeninges Breast Cancer HER2-positive Breast Cancer | City of Hope Medical Center | Non-Randomized | Single group assignment | None (Open Label) | Experimental: HER2-CAR T cells | HER2-CAR T cells: Intraventricular infusion, once weekly × 3 doses per cycle; additional cycles per investigator discretion if tolerated | 31/08/2018 | 19/02/2026 |
| NCT03220854 | Phase 2 Clinical Trial of Stereotactic Radiotherapy (SRT) and PD-1 or PD-L1 Inhibiting Therapy for Treatment of Advanced Solid Tumors After Disease Control on PD-1 or PD-L1 Inhibiting Therapy | Phase II | Completed | Solid Tumor | Virginia Commonwealth University | Non-Randomized | Parallel assignment | None (Open Label) | Experimental A: SBRT (body) irradiation only Experimental B: Patients receiving SBRT and SRS (body and brain) irradiation Experimental C: Patients receiving SRS (brain) irradiation only | Stereotactic radiotherapy: 18–60 Gy in 3–5 fractions over 1–2 weeks  Anti-PD-1: Continue prior PD-1 inhibitor (standard of care)  Anti-PD-L1: Continue prior PD-L1 inhibitor (standard of care) | 10/10/2017 | 22/09/2021 |
| NCT06514898 | MATCHPOINT - Medulloblastoma Adoptive T Cell Therapy, DC Vaccines, and Hematopoietic Stem Cells Combined With Immune checkPOINT Blockade | Phase I | Active, not recruiting | Recurrent Group 3 MB Recurrent Group 4 (Non-SHH/Non-WNT) MB | University of Florida | N/A | Single group assignment | None (Open Label) | Experimental: Adoptive Cellular Therapy (ACT) + PD-1 blockade with pembrolizumab | TTRNA-DC vaccines + GM-CSF: 10 ID vaccines total; 3 biweekly for priming, monthly for 2–3 cycles during T-cell expansion, 3 biweekly during T-cell engraftment; each with 150 µg GM-CSF.  TTRNA-xALT: Single IV infusion of T cells.  Td vaccine: Full booster IM at Vaccine #1; pretreatment at Vaccines #3, #5, #7, #9.  Autologous HSCs: Single IV infusion.  Pembrolizumab: IV PD-1 blockade starting with ACT, up to 2 years if tolerable and no progression. | 05/05/2025 | 01/12/2028 |
| NCT04164199 | An Open-Label, Multicenter, Long-Term Extension Study of Treatment With Tislelizumab, Pamiparib, and Other Investigational Agents in Patients With Advanced Malignancies | Phase III | Enrolling by invitation | Advanced Malignancies | BeiGene | Non-Randomized | Parallel assignment | None (Open Label) | A: Tislelizumab  B: Pamiparib  C: Sitravatinib  D: BGB-15025  E: Zanidatamab  G: Tislelizumab + Pamiparib  H: Tislelizumab + Sitravatinib  I: Tislelizumab + Ociperlimab  J: Tislelizumab ± BAT1706  K: Tislelizumab + Fruquintinib  L: Tislelizumab + BGB-A445  M: Tislelizumab + Surzebiclimab  N: Tislelizumab + BGB-15025  O: Tislelizumab + Lenvatinib  P: Tislelizumab + Zanidatamab  Q: Tislelizumab + LBL-007  R: Tislelizumab + Surzebiclimab + LBL-007 | Pamiparib, Sitravatinib, BGB-15025: Oral administration  Tislelizumab, Zanidatamab, Ociperlimab, Surzebiclimab: IV administration | 19/12/2019 | 2026-12 |
| NCT06639607 | Phase 1/2 Trial of PEP-CMV + Nivolumab for Newly Diagnosed Diffuse Midline Glioma/High-grade Glioma and Recurrent Diffuse Midline Glioma/High-grade Glioma, Medulloblastoma, and Ependymoma (PRiME II) | Phase I/II | Not yet recruiting | Diffuse Midline Glioma Diffuse Midline High-grade Glioma MB Ependymoma | Washington University School of Medicine | Non-Randomized | Parallel assignment | None (Open Label) | Phase I: Temozolomide + Nivolumab + PEP-CMV vaccine + Td booster  Stratum I: newly-diagnosed high-grade glioma or DMG Stratum II: recurrent/progressive HGG or DMG Stratum III: recurrent/progressive MB or EPN  Phase II: Temozolomide + Nivolumab + PEP-CMV vaccine + Td booster | Temozolomide: oral, 200 mg/m²/day, Days 1–5 (Cycle 1)  PEP-CMV vaccine: ID (½ each groin), Day 21, 35, 49 (Cycle 1), then every 28 days (subsequent cycles)  Td booster: IM at enrollment; pre-conditioning 6–24h before first vaccine  Nivolumab: IV, 3 mg/kg, Day 14 (Cycle 1) then every 14 days | 01/09/2025 | 30/09/2042 |
| NCT03615404 | A Phase 1 Trial of CMV RNA-Pulsed Dendritic Cells With Tetanus-Diphtheria Toxoid Vaccine in Pediatric Patients and Young Adults With WHO Grade IV Glioma, Recurrent Malignant Glioma, or Recurrent Medulloblastoma | Phase I | Completed | GBM Malignant Glioma MB Recurrent Pediatric GBM Multiforme Pediatric Brain Tumor, Recurrent Pediatric Brain Tumor | Gary Archer Ph.D. | N/A | Single group assignment | None (Open Label) | CMV-DCs with GM-CSF and Td (tetanus toxoid) | CMV-DCs with GM-CSF: autologous DCs loaded with pp65-flLAMP RNA, given with GM-CSF, ID  Td: preconditioning vaccine, ID, 6–24 h before first and subsequent CMV-DC vaccinations. | 05/10/2018 | 02/07/2020 |
| NCT00058370 | A Trial Of Radioimmunotherapy, Reduced-Dose External Beam Craniospinal Radiation Therapy With IMRT Boost, And Chemotherapy For Patients With Standard-Risk Medulloblastoma | N/A | Completed | Brain and CNS Tumors | Memorial Sloan Kettering Cancer Center | N/A | Single group assignment | None (Open Label) | Experimental: histologic proof of medulloblastoma | Cisplatin, Lomustine, Vincristine Sulfate: standard chemotherapy.  Iodine I-131 3F8: intrathecal administration.  Radiation therapy: reduced-dose craniospinal RT (1800 cGy) + primary site boost to 5400 cGy via IMRT. | 2003-02 | 2019-06 |
| NCT03389802 | Phase I Study to Evaluate the Safety and Tolerability of the CD40 Agonistic Monoclonal Antibody APX005M in Pediatric Subjects With Recurrent/Refractory Brain Tumors and Newly Diagnosed Brain Stem Glioma | Phase I | Active, not recruiting | GBM Multiforme High-grade Astrocytoma  CNS Primary Tumor  Ependymoma,  DIPG MB  NOS | Pediatric Brain Tumor Consortium | Non-Randomized | Sequential Assignment | None (Open Label) | Stratum 1: Recurrent or refractory primary malignant CNS tumor patients Stratum 2: Newly diagnosed DIPG patients | APX005M: IV every 21 days, up to 36 cycles or until progression/toxicity/death.; Stratum 1: 0.1 mg/kg starting, dose escalation/de-escalation to determine MTD; Stratum 2 (DIPG): start below RP2D from Stratum 1, adjust to RP2D. | 01/03/2018 | 30/09/2025 |
| NCT04167618 | A Phase I/II Dose-escalation and Expansion Cohort Trial of Intracerebroventricular Radioimmunotherapy Using 177Lu-DTPA-Omburtamab in Pediatric and Adolescent Patients With Recurrent or Refractory Medulloblastoma | Phase I/II | Terminated (Business priorities) | MB, Childhood | Y-mAbs Therapeutics | N/A | Single group assignment | None (Open Label) | Experimental: 177Lu-DTPA-omburtamab | 177Lu-DTPA-omburtamab: Intracerebroventricular administration for up to two cycles (Part 1) and up to five cycles (Part 2). | 30/09/2021 | 11/08/2022 |
| NCT04743661 | Phase 2 Study of Intraventricular Omburtamab-based Radioimmunotherapy for Pediatric Patients With Recurrent Medulloblastoma and Ependymoma | Phase II | Active, not recruiting | Recurrent MB Recurrent Ependymoma | Pediatric Brain Tumor Consortium | Non-Randomized | Parallel assignment | None (Open Label) | Stratum 1: Recurrent Medulloblastoma  Stratum 2: Recurrent Ependymoma | Irinotecan: 50 mg/m² IV, Days 1–5 per course (Stratum 1)  Temozolomide: 150 mg/m² PO, Days 1–5 per course (Stratum 1)  Bevacizumab: 10 mg/kg IV, Days 1 & 15 per course (last course: Day 1 only)  131I-Omburtamab: 50 mCi IV; Stratum 1: Days 8 & 36, Stratum 2: Days 1 & 29  Liothyronine: 25–50 µg PO/NG/G-tube daily, starting ≥7 days before and continuing ≥14 days after each 131I injection  SSKI: 7 drops PO/NG/G-tube daily, same schedule as liothyronine  Dexamethasone: 0.5–1 mg PO twice daily × 6 doses around each 131I injection (pre-, during, post-)  Antipyretic: e.g., acetaminophen 15 mg/kg PO, 1–3 h before 131I injection  Antihistamine: e.g., diphenhydramine 1 mg/kg IV, 1–3 h before 131I injection  Anti-emetic: e.g., ondansetron 0.25 mg/kg IV, 1–3 h before 131I injection | 04/04/2022 | 30/10/2030 |
| NCT02332889 | A Phase I/Pilot II Trial Combining Decitabine and Vaccine Therapy for Patients With Relapsed or Refractory Pediatric High Grade Gliomas, Medulloblastomas, and Central Nervous System Primitive Neuroectodermal Tumors (CNS PNETs) | Phase I/II | Terminated (Transition to a different immunotherapy strategy in the future at our institution) | Gliomas MB Neuroectodermal Tumors, Primitive | University of Louisville | N/A | Single group assignment | None (Open Label) | Experimental: Decitabine/Vaccine Therapy | Vaccine: DC thawed, washed, viability >70%, peptide-pulsed, administered by study physician.  Decitabine: 10 mg/m² IV over 1 h, Days 1–5 of Week 1.  Hiltonol: IM at the same site immediately after each DC vaccine. | 2015-04 | 2016-07 |
| NCT00014573 | Phase II Trial Of High Dose Cyclophosphamide, Cisplatin And Carmustine With Stem Cell Reconstitution Followed By Specific Cellular Therapy In Patients With Recurrent Or Refractory Brain Tumors | Phase II | Completed | Brain and CNS Tumors | Barbara Ann Karmanos Cancer Institute | N/A | Single group assignment | None (Open Label) |  | Biologicals: aldesleukin, autologous tumor cell vaccine, filgrastim, sargramostim, therapeutic autologous lymphocytes  Drugs: carmustine, cisplatin, cyclophosphamide, paclitaxel  Procedures: autologous bone marrow transplantation, peripheral blood stem cell transplantation, conventional surgery | 1998-08 | 2004-10 |
| NCT03838042 | INFORM2 Exploratory Multinational Phase I/II Combination Study of Nivolumab and Entinostat in Children and Adolescents With Refractory High-risk Malignancies (INFORM2-NivEnt) | Phase I/II | Recruiting | CNS Tumor Solid Tumor | University Hospital Heidelberg | N/A | Single Group Assignment (Phase I) | None (Open Label) | Experimental: Nivolumab and Entinostat | Nivolumab + Entinostat: Entinostat given alone for 1 week (priming), followed by combination with nivolumab. | 26/05/2020 | 30/06/2027 |

ACNU/ACNV: nimustine; ACT: adoptive cell therapy; Ara-C: cytarabine; ATRT: Atypical Teratoid/Rhabdoid Tumor; BCNU: carmustine; BID: twice daily; CAR: chimeric antigen receptor; CCNU: lomustine; CDDP: cisplatin; CMMRD: Constitutional Mismatch Repair Deficiency; CNS: central nervous system; CR: complete response; CTX: cyclophosphamide; DIPG: diffuse intrisinc pontine glioma; DL: dose level; DMG: diffuse midline glioma; GBM: glioblastoma; Gy: Gray; h: hours; HGG: high grade glioma; HSC: hematopoietic stem cell; ID: intradermal; IM: intramuscular; IMRT: Intensity-Modulated Radiation Therapy; IT: intrathecal; IV: intravenous; LP: lumbar puncture; MB: medulloblastoma; mo: month; MTD: maximum tolerated dose; MTX:methotrexate; N/A: not assigned; NE: non evaluable; NG: nasogastric; NOS: not otherwise specified; PD: progressive disease; POM: pomalidomide; PNET: primitive neuroectodermal tumor; PO: *per os* (oral); PR: partial response; q28d: every 28 days; q2w: every 2 weeks; q3w: every 3 weeks; RP2D: recommended phase 2 dose; RTH: radiotherapy; SD: stable disease; UN: unknown; VCR: vincristine; VP-16: etoposide; wk: week; yrs: years.
